# Penile coital injuries in men decline after circumcision: Results from a prospective study of recently circumcised and uncircumcised men in western Kenya

**DOI:** 10.1371/journal.pone.0185917

**Published:** 2017-10-10

**Authors:** Nelli Westercamp, Supriya D. Mehta, Walter Jaoko, Timothy A. Okeyo, Robert C. Bailey

**Affiliations:** 1 Division of Epidemiology and Biostatistics, School of Public Health, University of Illinois at Chicago, Chicago, Illinois, United States of America; 2 Department of Medical Microbiology, University of Nairobi, Nairobi, Kenya; 3 Nyanza Reproductive Health Society, Kisumu, Kenya; Cardiff University, UNITED KINGDOM

## Abstract

**Background:**

Penile coital injuries are one of the suggested mechanisms behind the increased risk of HIV among uncircumcised men. We evaluated the prevalence and correlates of self-reported penile coital injuries in a longitudinal community-based cohort of young (18–24 years old), newly circumcised and uncircumcised men in Western Kenya.

**Methods:**

Self-reported penile coital injuries were assessed at baseline, 6, 12, 18 and 24 months of follow-up, and were defined as scratches, cuts or abrasions during sex, penile soreness during sex, and skin of the penis bleeding during sex. Associations between penile coital injuries, circumcision, sexual satisfaction, and other covariates were estimated with mixed effect models.

**Results:**

Between November 2008 and April 2010 3,186 participants were enrolled (1,588 into circumcision group and 1,598 as age-matched controls). Among 2,106 (66%) participants sexually active at baseline, 53% reported any penile injury, including 44% scratches, cuts or abrasions; 32% penile pain/soreness; and 22% penile bleeding. In multivariable modeling, risk was lower for circumcised men than uncircumcised men for scratches, cuts and abrasions (aOR = 0.39; 95% CI 0.34–0.44); penile pain/soreness (aOR = 0.58; 95% CI 0.51–0.65), penile bleeding (aOR = 0.53; 95% CI 0.46–0.62), and any penile coital injuries (aOR = 0.47; 95%CI 0.42–0.53). Other significant risk factors included increasing age, history of STIs and genital sores, and multiple sex partners, while condom use was protective. Coital injuries were significantly associated with lower levels of sexual satisfaction in longitudinal analyses (scratches, cuts or abrasions: aOR = 0.87, 95% CI: 0.76–0.98; penile pain/soreness: aOR = 0.82, 95% CI: 0.72–0.93; and penile bleeding: aOR = 0.65, 95% CI: 0.55–0.76).

**Conclusions:**

Self-reported penile coital injuries were common and decreased significantly following circumcision. Improving sexual experience through the removal of a potential source of sexual discomfort may resonate with many men targeted for circumcision services. The role of penile coital injuries in sexual satisfaction, HIV, HSV-2, and as a motivator for seeking circumcision services should be explored further.

## Introduction

Three randomized control trials (RCT) of male circumcision (MC) for HIV prevention in Kenya [1], Uganda [2], and South Africa [3], have demonstrated the protective effect of male circumcision against heterosexually-acquired HIV infection in men beyond any reasonable doubt [4, 5]. While the exact biological mechanism by which MC affords this protection is not known [6], there are a number of plausible explanations based on the cellular composition and environment of the inner foreskin. The earliest hypotheses concerned the gross anatomy of the uncircumcised penis, including the feasibility of potentially infectious secretions being trapped in facilitating conditions beneath the foreskin [4, 7] and the increased surface area of the inner foreskin [8, 9]. With the recognition that ulcerative STIs and other causes of genital tract inflammation increase the risk of HIV infection [10, 11], the association between these infections and circumcision offers additional possible explanation [7, 12–14]. Histologic examination and specific immune responses of the foreskin, and differences in the penile microbiome of circumcised and uncircumcised men offers another set of mechanisms [15–18]. Like most biologic mechanisms, the protective effect of circumcision almost certainly represents a complex system incorporating multiple explanatory factors [19–22].

One possible foreskin-associated HIV risk factor that is often mentioned, but that has not received attention in empirical research, is the perception that preputial mucosa is comparatively fragile and prone to injury during intercourse [7, 8, 23–25]. One difficulty in determining the role of intercourse-associated mechanical injury to the penis in HIV infection is a lack of consistent terminology or operational assessment. When discussed, mechanical penile injuries are often referred to as minor epithelial disruptions, penile trauma, traumatic lesions, or most recently as penile coital injuries, clarifying an integral sexual component [7, 12, 25–28].

Recent research in men participating in the Kenya RCT has shown that penile coital injuries are more commonly reported among uncircumcised men [27], may be an important non-STI cause of genital ulcer disease (GUD) [29], and increase the risk of *Neisseria gonorrhoeae* [30] and HIV [31]. High pre-circumcision baseline prevalence of coital injuries was observed in a circumcision cohort in the Dominican Republic, with a subsequent decline following circumicsion [32]. Outside of these populations there is little information available on coital injury prevalence, associated factors, or related disease susceptibilities. Similarly, the potential association between the coital injuries and sexual satisfaction remains largely unexplored.

To increase the understanding of the prevalence and correlates of penile coital injuries and the potential effect on sexual satisfaction in a more general population, we evaluated three types of self-reported penile coital injuries in a longitudinal community-based cohort of newly circumcised and uncircumcised men in Nyanza Province, Kenya.

## Methods

### Study design and participants

The study took place between November 2008 and January 2012 in one urban (Kisumu East) and two rural (Nyando and Kisumu West) sub-counties in western Kenya. Additional study details, sample description and the outcomes of the behavioral risk compensation assessment have been previously described by Westercamp et al. [33]. To participate, men had to be uncircumcised, 18 to 35 years old, live within the study area, and have no plans to relocate within the next 2 years. Eligible men self-selected into the circumcision group by seeking circumcision services at a voluntary medical male circumcision (VMMC) clinic within the study area, and were recruited before risk-reduction counseling and the circumcision procedure itself were completed. A control group was recruited from the community surrounding each VMMC clinic site and frequency-matched to age and residence (community) of the circumcision group. The controls were men who declined the opportunity to become circumcised before enrollment in the study.

Participants provided written informed consent in their language of choice (English, Dholuo or Kiswahili) and were offered 200 Kenyan shillings (about $2.50) for each study visit to cover travel expenses and loss of income. Ethical approval was obtained from the Kenyatta National Hospital Ethics and Research Committee and the Institutional Review Board of the University of Illinois at Chicago.

### Study procedures

Study participants completed a detailed sexual history and behavioral questionnaire and had their circumcision status visually confirmed by specially trained research assistants at each study visit: baseline, and 6, 12, 18, and 24-month follow-up visits. All participants were uncircumcised at baseline interview. Men who intended to become circumcised proceeded through the normal VMMC clinic flow following the Kenyan government VMMC guidelines.

Study questionnaires were administered through audio computer-assisted self-interview (ACASI) modules, developed in English, Dholuo, and Kiswahili. An equivalent paper-based questionnaire was used at participant request or in cases of power outage at study facilities (~30% of all questionnaires). The questionnaire instrument included items related to socio-demographic characteristics, sexual behaviors, history of STIs, general reproductive health, and sexual function and satisfaction including a set of questions addressing penile coital injuries. All data were self-reported and no biological samples were taken.

### Statistical analyses

Self-reported penile coital injuries were determined by asking men, “In the past 6 months …: 1) how often during sex did the skin of your penis get scratches, cuts, or abrasions? (labeled as “scratches/cuts/abrasions” in the analyses); 2) how often during sex did your penis get sore? (labeled as “pain/soreness” in the analyses); and 3) how often during or after sex did the skin of your penis bleed? (labeled as “penile bleeding” in the analyses)” The response set was dichotomized for analysis as ever (by grouping always, often, sometimes or rarely) vs. never. To facilitate comparison, the penile injury assessment was the same as used by Mehta *et al*. in their 2010 evaluation of penile coital injuries in the Kenyan RCT and, to the extent possible, variables selected for analysis were kept consistent [27]. These variables included: condom use at last sex, preference for dry sex, applying substances on penis before sex, self-reported STIs in the past 6 months, genital hygiene after sex and circumcision status. Demographic (i.e., age, marital status, education, employment and ethnicity) and behavioral (i.e., number of partners in the past 6 months) variables were also included in the analysis. Circumcision status was treated as a time-varying covariate to accommodate crossovers. Pearson χ2 tests for categorical variables and Kolmogorov-Smirnov two-sample test for non-normally distributed continuous variables were used to assess baseline differences between circumcision and control group participants.

Because reports of abrasions, scratches or cuts to the penis may represent a misclassification of genital ulcers related to infectious etiologies, we identified men with more chronic GUD symptoms by asking, “Have you experienced any sores on or around genitals in the past 6 months.” A visual genital exam was done to confirm circumcision status, but did not assess genital health or STI signs.

Sexual satisfaction was assessed by asking participants, “In the past 6 months, how would you generally rate your satisfaction with sexual intercourse?” The responses were dichotomized for analysis as satisfied (by grouping very satisfied and satisfied) and dissatisfied (by grouping very dissatisfied, dissatisfied, and unsure).

We compared penile coital injuries reported by circumcised and uncircumcised participants using random intercept mixed-effect models for binary outcomes to account for within-subject correlation due to repeated measures. Penile coital injuries were modeled for each participant as the linear slope over time assessed in 6-month intervals between the baseline and the 24-month follow-up time. The model included circumcision group as a binary variable and a group by time interaction to allow for varying trajectories between the two groups over time. To quantify the differences between circumcised and uncircumcised men over 24 months, we estimated odds ratios (OR) for group effect through mixed-effect models by excluding the circumcision by time interaction. All analyses were restricted to men sexually active in the 6 months preceding the interview. Covariates were selected for inclusion in multivariable models based on univariate analyses (p<0.05) and previous literature. All variables, except for age, education, and ethnicity, were time-varying covariates. Final model selection was done using backwards elimination with study time and age included in all models. Sexual satisfaction was modeled for each participant as the linear slope over time assessed in 6-month intervals between the baseline and the 24-month follow-up time. The primary independent predictors were the three types of penile coital injuries, with adjustment for time, circumcision status, age, education, employment, number of partners in the past 6 months, and reports of genital sores and STIs in the past 6 months. To ensure the comparability of our results with studies using other modeling approaches, population-averaged odds ratios were calculated by transforming our subject-specific regression estimates as described by Hu et al. [34]. All presented odds ratios are population-averaged. Statistical analyses were performed using SAS v9.2 [35] with the NLMIXED procedure for mixed-effect modeling.

## Results

### Sample description

Between November 2008 and April 2010, 3,186 of 3,825 (97%) of eligible men agreed to participate in the study. By design, study groups were equally sized (1,588 circumcision group; 1,598 control group). Approximately 5% of both groups (79/1588: circumcision and 74/1598: controls) were lost to follow-up after the baseline assessment and were excluded from longitudinal analyses. Men not returning for any follow-up were less likely to be Luo (p = 0.03) and less likely to have ever had sex (p = 0.01). Follow-up rates were 70% (6-months), 81% (12-months), 82% (18-months), and 84% (24-months), with similar loss to follow-up for the two groups. There were differences between the self-selected groups, with men in the circumcision group more likely to have a secondary or higher education, be unemployed, and single than the control group (Table 1); and no differences in other demographic or behavioral characteristics.

**Table 1:** Baseline characteristics of study participants.

	Circumcision group (N = 1 588)	Control group (N = 1 598)	Overall (N = 3 186)	p-value
**Demographic characteristics**				
**Age, mean (IQR, range)**	20 (19–24; 18–35)	20 (19–24; 18–35)	20 (19–24; 18–35)	0.08
**Ethnic group**				0.01
Luo	1547 (97%)	1585 (99%)	3132 (98%)	
**Educational level**				0.01
Primary and less	354 (22%)	488 (31%)	842 (26%)	
Any secondary	955 (60%)	882 (55%)	1837 (58%)	
Any post-secondary	266 (17%)	306 (13%)	472 (15%)	
Unsure / Refused to answer	13 (1%)	22 (1%)	35 (1%)	
**Employment status**				0.01
Employed	421 (27%)	584 (37%)	1005 (32%)	
**Marital status**				0.01
Single, without live-in partner	1055 (66%)	956 (60%)	2011 (63%)	
Single, with live-in partner	220 (14%)	203 (13%)	423 (13%)	
Married, living with wife	271 (17%)	401 (25%)	672 (21%)	
Married, not living with wife	42 (3%)	38 (2%)	80 (3%)	
**Sexual history**				
**Ever had sex**				0.13
Yes	1382 (87%)	1419 (89%)	2801 (88%)	
No	206 (13%)	179 (11%)	385 (12%)	
**Age (years) at first sex (IQR; range; N)**	16 (15–18; 9–30; 1380)	16 (15–18; 9–29; 1417)	16 (15–18; 9–30; 2797)	0.66
**Sexual intercourse in past 6 months (sexually active only)**				0.54
Yes	1032 (75%)	1074 (76%)	2106 (75%)	
No	350 (25%)	345 (24%)	695 (25%)	
**Number of partners in past 6 months (sexually active only)**				0.13
None	350 (25%)	345 (24%)	960 (34%)	
One	502 (36%)	564 (40%)	1008 (36%)	
2+	393 (29%)	398 (28%)	786 (28%)	
Unsure / Refused to answer	137 (10%)	112 (8%)	147 (2%)	
**Lifetime number of partners**	3 (2–6; 1–552; 1193)	3 (2–6; 1–122; 1270)	3 (2–6; 1–552; 2463)	0.73
**Penile coital injuries in the past 6 months (sexually active in last 6 months, N = 2,045)**				
**Reported penile coital injuries in the past 6 months:**				
Scratches/cuts/abrasions	477 (47%)	428 (41%)	905 (44%)	0.01
Penile pain/soreness	343 (34%)	321 (31%)	664 (32%)	0.15
Penile bleeding	234 (23%)	211 (20%)	445 (22%)	0.12
Any penile coital injury	562 (56%)	518 (50%)	1080 (53%)	0.01

Sample sizes vary in questions based on past or recent sexual activity. Data are median (IQR; range; n) for continuous data, or n (%) for categorical data. P values are based on Kolmogorov-Smirnov two-sample test for non-normally distributed continuous data and chi-square for categorical data.

### Baseline penile coital injuries

Among the 2,106 (66%) participants sexually active in the 6 months prior to baseline, 2,045 (97%) answered the questions about penile coital injuries (Table 1). In total, 1,080 (53%) reported any penile injury, including 44% reporting scratches, cuts, or abrasions; 32% penile pain/soreness; and 22% penile bleeding. Overall, 259 (13%) of men reported all three types of coital injuries, 413 (20%) two types, and 408 (20%) reported a single type of penile coital injury.

At baseline, men who selected circumcision were significantly more likely to report a recent history of penile coital scratches, cuts, or abrasions compared to men who were initially not planning to become circumcised (47% vs. 41%, p = 0.007; Table 1). Reports of penile pain/soreness and penile bleeding did not differ by circumcision intent at baseline (Table 1). Combined, a greater proportion of those who selected circumcision reported at least one type of penile coital injury compared to those who selected to remain uncircumcised (56% vs. 50%; p = 0.01; Table 1).

### Penile coital injuries by circumcision status

**Fig 1:**
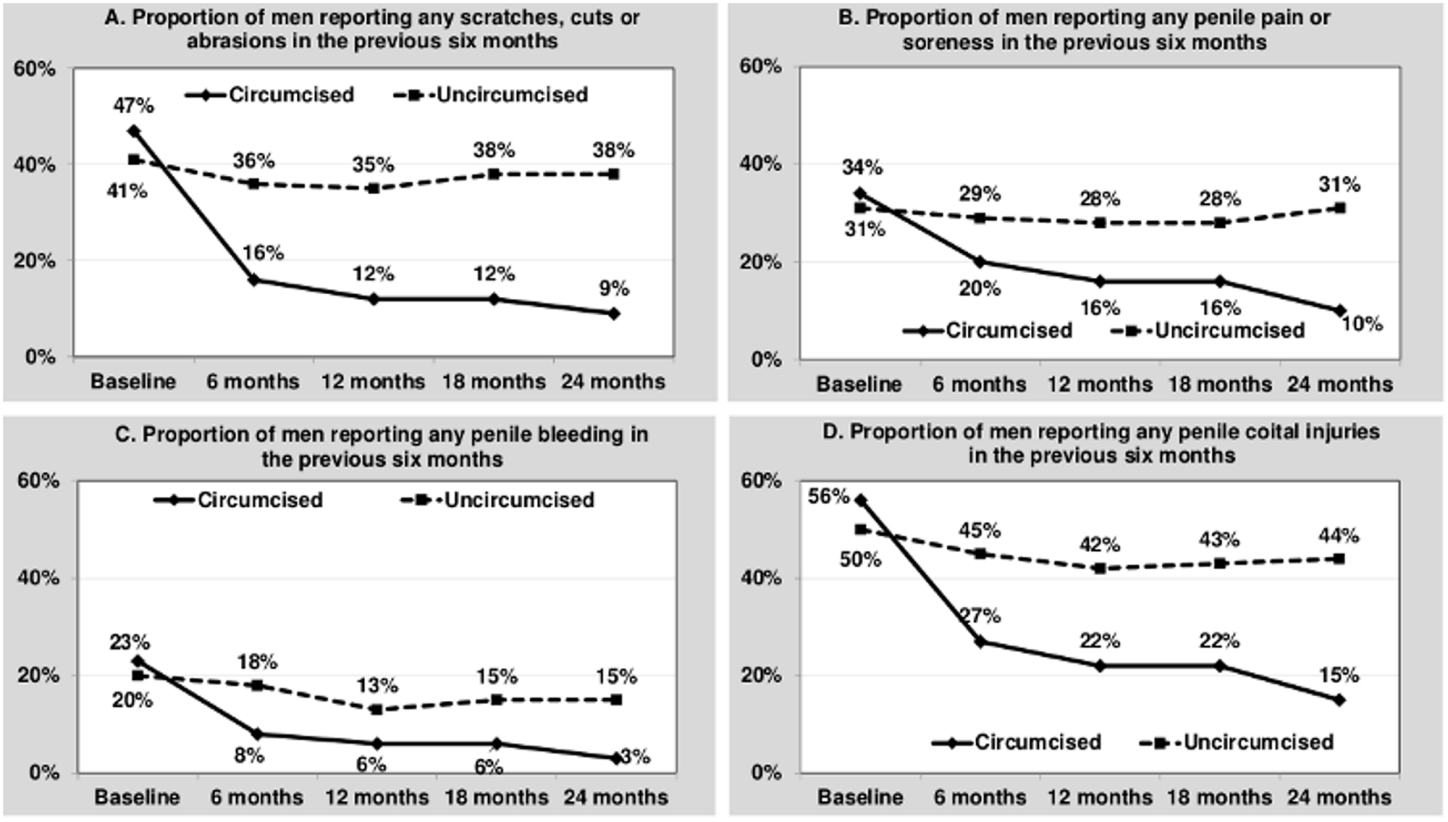
Observed self-reported penile coital injury by circumcision status over time. Notes: There was a significant decline with time for all measures of penile coital injuries, except for scratches/cuts/abrasions (A) and pain/soreness (B) in uncircumcised men.

Over 24 months of follow-up, reports of any penile coital injuries following circumcision declined from 56% (pre-circumcision baseline) to 15%, a 73% relative decrease. Significant decline was noted for each component individually: scratches/cuts/abrasions (81% decrease), penile pain/soreness (71% decrease), and penile bleeding (87% decrease). All declines were evident at 6-months post-procedure and were sustained throughout the follow-up period (Fig 1). Among the uncircumcised group, no decline was noted in cuts, scratches, abrasions or post-coital penile soreness, while a 25% decline in coital penile bleeding from 20% of men at enrollment to 15% at 24 months was observed.

Longitudinal analysis using mixed-effect models revealed the presence of group by time interaction (p<0.0001) for all measures of penile coital injuries indicating different patterns of change in injuries over time for circumcised and uncircumcised men. When stratified by circumcision status, decline from pre-circumcision baseline over time was significant for circumcised men across all three types of coital injuries (Table 2). For uncircumcised men, statistically significant decline of lower magnitude was observed only for penile bleeding and for the combined measure of any coital injury (Table 2).

**Table 2:** Longitudinal changes in penile coital injuries over 24 months of follow up by circumcision status: the results of mixed effect models (stratified by circumcision status, unadjusted for other covariates).

	Stratified analysis					
	Circumcised			Uncircumcised		
	Estimate	SE	p	Estimate	SE	p
**Scratches, cuts and abrasions**						
Change over time (visit #)	-0.719	0.037	0.01	-0.031	0.026	0.24
**Penile pain/soreness**						
Change over time (visit #)	-0.451	0.032	0.01	-0.015	0.027	0.58
**Penile bleeding**						
Change over time (visit #)	-0.717	0.05	0.01	-0.121	0.032	0.01
**Any penile injuries**						
Change over time (visit #)	-0.609	0.031	0.01	-0.082	0.025	0.01

In addition to exploring the differential changes in coital injuries in each group over time (Table 2), we reran the models without the group by time interaction term to be able to quantify the association between coital injuries and circumcision for the duration of the study. By removing the interaction term from the model, the estimates of the effect of circumcision were adjusted for time, but not for the differential dynamics of time for each group. As a result, OR comparing circumcised to uncircumcised men over 24 months of follow up was 0.49 (95% CI: 0.44–0.55) for any penile coital injury. By type, odds ratios were 0.41 (95% CI: 0.37–0.47) for scratches, cuts and abrasions, 0.59 (95% CI: 0.52–0.66) for penile pain/soreness, and 0.54 (95% CI: 0.47–0.62), for penile bleeding.

### Penile coital injuries and genital sores

At baseline, 186 (9%) of sexually active men reported genital sores in the past 6 months. Compared to men not reporting sores, a higher proportion of men with genital sores were seeking circumcision, had only primary education, were employed, married, had multiple partners in the past 6 months, had unprotected sex, preferred dry sex, and reported STIs in the past 6 months. Following circumcision, the proportion of men reporting genital sores declined from 11% (baseline, among men selecting circumcision) to 2% at 24-months. Among the uncircumcised men, no change in the prevalence of genital sores was observed: 8% at baseline to 8% at 24 months. At baseline, 14% of men reporting any penile coital injuries also reported genital sores (14% of men reporting scratches, cuts, or abrasions, 19% reporting penile pain/soreness, and 14% with penile bleeding). Conversely, over 81% of men reporting a genital sore at baseline also reported a concomitant penile coital injury (Table 3), including 68% reporting scratches, cuts, or abrasions, 66% with penile pain/soreness, and 34% with bleeding.

**Table 3:** Prevalence of reported coital injuries across time-varying covariates significantly associated with coital injuries in univariate analyses.

	Baseline, n(%)	6 months, n(%)	12 months, n(%)	18 months, n(%)	24 months, n(%)
Circumcision status*					
Circumcised	562/1008(56%)	200/732 (27%)	202/929(22%)	238/1071(22%)	180/1199(15%)
Uncircumcised	518/1037(50%)	401/888(45%)	390/920(42%)	386/892(43%)	375/848 (44%)
**Marital status** *					
Single	569/1143(50%)	270/852(32%)	295/976(30%)	271/1001(27%)	1236/1008(23%)
Married or cohabitating	511/902(57%)	331/768(43%)	297/873(34%)	353/962(37%)	319/1039(31%)
**Condom use at last sex** *					
No condom used	572/1001(57%)	321/736(44%)	324/861(38%)	321/861(37%)	280/912(31%)
Condom used	435/892(49%)	252/800(32%)	243/917(27%)	290/1064(27%)	263/1106(24%)
**Number of partners in the past 6 months** *					
One	444/1012(44%)	257/804(32%)	276/1030(27%)	311/1171(27%)	328/1353(24%)
Two or more	501/756(66%)	294/652(45%)	275/686(40%)	279/695(40%)	214/643(33%)
**How long until washed penis after last time had sex** *					
One hour or less	383/783(49%)	232/662(35%)	236/798(30%)	261/917(29%)	275/1085(25%)
More than one hour	697/1262(55%)	369/958(39%)	356/1051(34%)	363/1047(35%)	280/962(29%)
**Applied substances to penis before sex in the past 6 months** *					
No	885/1719(52%)	516/1407(37%)	507/1653(31%)	543/1776(31%)	493/1874(26%)
Yes	85/114(75%)	46/89(52%)	45/75(60%)	43/79(54%)	37/83(45%)
**Preference for dry sex**					
Prefers dry sex	395/749(53%)	231/614(38%)	232/662(35%)	260/727(36%)	219/695(36%)
Prefers wet sex or no opinion	575/1084(53%)	331/881(38%)	320/1066(30%)	326/1128(29%)	311/1262(25%)
**STIs in the past 6 months** *					
No	885/1715(52%)	525/1440(37%)	519/1675(31%)	548/1799(31%)	500/1909(26%)
Yes	88/118(75%)	38/56(68%)	33/49(67%)	37/54(69%)	29/45(64%)
**Genital sores in the past 6 months** *					
No	930/1859(50%)	532/1533(35%)	519/1750(30%)	540/1860(29%)	478/1950(25%)
Yes	150/186(81%)	69/87(79%)	73/99(74%)	84/104(81%)	77/97(79%)

Circumcision status at baseline is by enrollment group; at follow up by actual status;

* Significant baseline difference

In newly circumcised men reporting genital sores, concurrent report of any penile coital injuries did not significantly decline over follow-up (pre-circumcision baseline 81% to 79% at 24-months; p = 0.22). In men with no history of genital sores, however, reports of penile coital injuries declined from 53% (pre-circumcision baseline) to 14% (p<0.001; ~80% relative decrease) over the 24-months following circumcision. Therefore, following circumcision we observe a decline in reports of recent genital sores and decline in penile coital injuries, but the latter only when men have not also experienced penile sores, indicating differential trends in prevalence of coital injuries among circumcised men with and without genital sores, as confirmed by the significant interaction term (p = 0.002).

**Fig 2:**
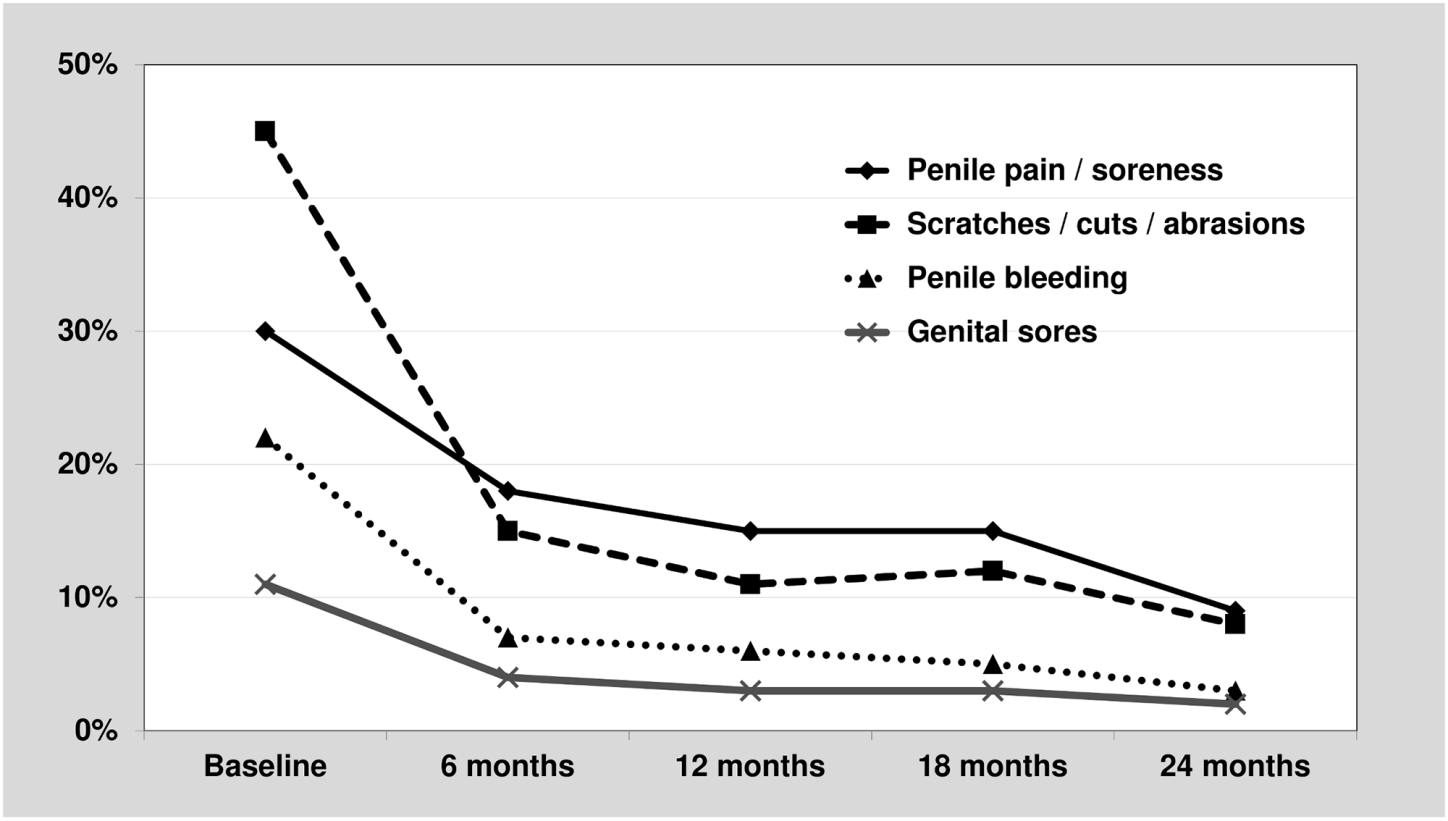
Genital sores and type of coital injury among those without genital sores over time (circumcised men only).

Considering any report of genital sores separately from that of penile coital injuries, the protective effect of circumcision on both is evident (Fig 2). Unadjusted ORs for the effect of circumcision over the duration of follow up were 0.67 (95% CI: 0.56–0.79) for recent history of genital sores. By type of coital injury, excluding those with genital sores, ORs by circumcision status were 0.43 (95%CI: 0.38–0.48) for scratches, cuts and abrasions, 0.61 (95%CI: 0.54–0.69) for penile pain/soreness, and 0.58 (95%CI: 0.50–0.67) for bleeding. Excluding men with genital sores did not greatly change (<10%) the magnitude of the association, therefore our multivariable analyses included men with and without genital sores and reports of genital sores were evaluated as a covariate for each type of penile coital injuries.

### Other covariates and multivariable models

The distribution over time of penile coital injuries across time-varying covariates significant in the univariate analyses is shown in Table 3. These covariates were included in the multivariable modeling to assess their association with penile coital injuries. The statistically significant covariates were retained in the final models and are shown in Table 4. In multivariable modeling, adjusted for other covariates, circumcised men were more than 50% less likely than those uncircumcised to report any penile coital injury in the last 6 months (aOR = 0.47; 95%CI 0.42–0.53). This protection was greatest against penile cuts, scratches, or abrasions for which the difference reached 60% (aOR = 0.39; 95%CI 0.34–0.44; Table 4). Factors independently associated with increased risk of each type penile coital injury were the application of substances (lubricants) to the penis before sex (aORs ranged 1.94 to 2.29), increasing age (aOR = 1.03 for each added year), history of STI in the last 6 months (aORs ranged 1.66 to 2.48), reporting genital sores in the last 6 months (aORs ranged 2.60 to 4.27), and multiple partners in last 6-months (aORs ranged 1.38 to 1.58). Condom use (aORs ranged 0.73 to 0.75) and time remained protective for penile coital injuries. While these factors were significantly associated with penile coital injuries, they did not act as confounders to the effect of circumcision, as seen by comparing the crude and adjusted ORs for the three types of penile coital injuries, though adjusting for covariates did attenuate the magnitude of change over time. Out of the variables differentiating men selecting circumcision and controls at baseline (i.e., education, employment, ethnicity and marriage), only marriage was a significant risk factor for coital injuries in the univariate analyses, but not in the multivariable analyses. Likewise, post-coital hygiene, and preference for dry sex were not associated with any of penile coital injury measures.

**Table 4:** Results of the multivariable mixed effect models for risks of penile coital injury over time (N = 2781).

	Scratches/cuts/ abrasions	Penile pain /soreness	Penile bleeding
	OR (95% CI)	OR (95% CI)	OR (95% CI)
Circumcision status*			
Uncircumcised	Reference	Reference	Reference
Circumcised	0.39 (0.34–0.44)	0.58 (0.51–0.65)	0.53 (0.46–0.62)
**Age (continuous)**	1.03 (1.02–1.05)	1.03 (1.01–1.04)	1.02 (1.00–1.04)
**Condom use at last sex**			
No condom used	Reference	Reference	Reference
Condom used	0.74 (0.67–0.83)	0.73 (0.65–0.81)	0.75 (0.65–0.86)
**Number of partners in the past 6 months**			
One	Reference	Reference	Reference
Two or more	1.48 (1.35–1.63)	1.38 (1.26–1.52)	1.58 (1.41–1.78)
**Ever applied substances to penis before sex**			
No	Reference	Reference	Reference
Yes	1.94 (1.55–2.42)	2.08 (1.66–2.60)	2.29 (1.79–2.93)
**Self-reported genital sores in the past 6 months**			
No	Reference	Reference	Reference
Yes	3.65 (2.99–4.46)	4.27 (5.09–6.39)	2.60 (2.10–3.20)
**Self-reported STIs in the past 6 months**			
No	Reference	Reference	Reference
Yes	1.66 (1.29–2.15)	2.48 (1.92–3.20)	2.16 (1.65–3.84)
**Time (visit)**			
Baseline	Reference	Reference	Reference
6 months	0.43 (0.37–0.49)	0.77 (0.66–0.89)	0.60 (0.50–0.71)
12 months	0.38 (0.32–0.44)	0.67 (0.57–0.78)	0.45 (0.37–0.54)
18 months	0.43 (0.37–0.49)	0.66 (0.57–0.76)	0.48 (0.40–0.58)
24 months	0.39 (0.34–0.45)	0.59 (0.51–0.69)	0.39 (0.33–0.48)

* Circumcision status at baseline is by enrollment group; at follow up by actual status.

All variables are time varying, except for age at baseline

### Penile coital injuries and sexual satisfaction

Sexual satisfaction and its association with male circumcision are examined in depth elsewhere [36]. Here we explore the associations between male circumcision and sexual satisfaction, in the context of penile coital injuries. At baseline, the proportion of men reporting being satisfied with sexual intercourse was lower among men reporting penile coital injuries compared to those not reporting coital injuries: 67% of men with scratches, cuts, and abrasions were satisfied with intercourse compared to 74% without scratches, cuts, and abrasions (p = 0.001); 65% with penile pain/soreness vs. 74% without penile pain/soreness (p<0.001); and 61% with penile bleeding vs. 74% without penile bleeding (p<0.001). Adjusted for time, circumcision status, age, education, employment, number of partners in the past 6 months, and reports of genital sores and STIs in the past 6 months, penile coital injuries remained significantly associated with lower levels of sexual satisfaction in longitudinal analyses (scratches, cuts and abrasions: aOR = 0.87, 95% CI: 0.76–0.98; penile pain/soreness: aOR = 0.82, 95% CI: 0.72–0.93; and penile bleeding: aOR = 0.65, 95% CI: 0.55–0.76). After adjusting for penile coital injuries, history of STIs and genital sores, and other factors, circumcision status remained marginally associated with increased sexual satisfaction (aOR = 1.13, 95% CI: 0.99–1.28).

## Discussion

In our study comparing recently circumcised men and uncircumcised men in western Kenya, we confirm previous observations that circumcised men are less likely to report penile coital injuries, with significantly decreased risk observed as early as 6 months after surgery and decreasing further over 24 months [27, 32]. Factors other than circumcision associated with penile coital injury included increasing age, increasing number of sexual partners, application of substances to the penis before sex, and self-reported history of STIs. The differences in prevalence of penile coital injuries at the baseline among otherwise similar groups indicates that it is likely that men reporting penile coital injuries were more likely to seek VMMC services. This study is the first to identify an association between self-reported penile coital injuries and decreased sexual satisfaction.

We found that coital-related scratches, cuts, and abrasions to the penis among young, uncircumcised, sexually active men were common (44%). This prevalence is comparable to the 48% observed by Mehta and colleagues in this same geographical area [27], but higher than the 34% found in a 1997 cross-sectional study in eastern Uganda [37], and lower than 61% reported in the cohort of circumcised men in the Dominican Republic before circumcision [32]. Several explanations for these differences are possible including: differences in the behavioral risk profile of the study samples, culturally specific sexual practices that increase the risk of coital injuries, circumcision status misclassification, and misclassification of GUD and injuries.

Reduction in GUD is a highly likely mechanism for at least some portion of the protective effect of MC against HIV [7, 25, 38], with a 41%-48% reduction in GUD following the procedure observed in recent studies [12, 28]. While not directly comparable due to methodological differences in the assessment of penile sores and genital ulcers, the 33% decline in the likelihood of self-reported genital sores with circumcision observed in our study is in the lower range of effect sizes reported. Moreover, men reporting genital sores were more likely to report penile coital injuries, suggesting an additional risk of injury with genital sores or some overlap between the symptoms of genital sores and of penile coital injuries. Unlike other studies, we found no significant reductions (p = 0.22) in penile injuries over time among men reporting genital sores after becoming circumcised. This finding could possibly be a result of unadjusted confounding due to the ambiguity in our measurements of genital sores and should be evaluated further.

Recent research has observed that approximately 40% to 60% of genital ulcers are not explained by STI etiologies [12, 28, 29]. Coital injuries may play a role in the formation of these unexplained ulcers through facilitating infections by other, non-sexually transmitted, pathogens [12, 28, 29, 38]. In stratified analysis, we observed that recent genital sores were reported by14% of men with penile coital injuries. This leaves a great majority of penile coital injuries that are likely attributable to mechanical disruption or factors unrelated to preexisting GUD. Future studies should include questions designed to differentiate between coital injuries and GUD or include clinical examination to confirm current injuries or sores.

This is the first study to identify an association between penile coital injuries and decreased sexual satisfaction. This association remained significant over time and for all three types of coital injuries, independent of age, circumcision status, level of sexual activity or other causes of genital discomfort, such as GUD and other STIs. In our study, men who were seeking circumcision were more likely to report penile coital injuries at the baseline. As described in an in-depth analysis of male circumcision and sexual satisfaction based on the same cohort of men [36], men self-selecting into the circumcision group were also more likely to report lower levels of sexual satisfaction at baseline. While we did not assess this directly, it is possible that lower sexual satisfaction associated with coital injuries was one of the motivating factors behind their decision to become circumcised.

Our findings are subject to several limitations. Data on penile coital injuries, genital sores and STIs were based on self-report with no corresponding clinical exam. However, similarities between our results and those from the RCT of male circumcision in Kisumu support the generalizability of our results: we used the same questions as the trial in a sample different in demographic, geographic, and other characteristics. Because study participants self-selected for enrollment and group assignment, it is possible that the motivation to become circumcised represents fundamental differences between study groups. Behavior, sexual history, and sexual satisfaction were self-reported, and they are subject to social desirability and recall biases, although we have limited these biases through the use of ACASI and experienced study staff with training in sensitive face-to-face interview techniques [39–42]. Sexual satisfaction was assessed using questions similar to those used in other studies [43], but we did not use validated instruments. Lastly, our study did not assess the likely mechanisms leading to coital injuries. This is an important aspect in determining how circumcision may be protecting men and ultimately in developing additional interventions and messaging.

## Conclusion

Penile coital injuries have logical and observable associations with increased risk of HIV and STI infection [25, 38, 44, 45]. While their prevention may be important in that regard alone, the potential motivational force for circumcision may also be of value. In our study we found that men reporting penile coital injuries were more likely to seek VMMC services and observed a significant decline in coital injuries following circumcision. Further, men seeking circumcision services had consistently lower levels of pre-procedure sexual satisfaction [46] and those with penile coital injuries had lower levels of sexual satisfaction both at the baseline and across follow-up. Improvement of the sexual experience through the removal of a potential source of sexual discomfort may resonate with a significant portion of men targeted for VMMC [25, 38, 44, 45, 47]. The accumulation of evidence indicating an independent role of penile coital injuries in decreased sexual satisfaction, HIV [31], and HSV-2 [30], merits comprehensive study to clinically and etiologically define penile coital injuries for potential intervention targets.

## Supporting information

S1 File Study questionnaires (English, Dholuo, and Kiswahili). (ZIP)

S2 File Data repository. (CSV)
