# Supplementary material for: Penile coital injuries in men decline after circumcision: Results from a prospective study of recently circumcised and uncircumcised men in western Kenya
Source: PLoS One. 2017 Oct 10;12(10):e0185917. doi: 10.1371/journal.pone.0185917 (PMC5634596; doi:10.1371/journal.pone.0185917)
Supplement: S1 File — (ZIP) [file pone.0185917.s001.zip › SHABS FORM 02 - Kiswahili v2.pdf]

SHABS	**ORODHA YA MASWALI KUHUSU MIENENDO**<br>**Version 2 / February 06, 2008**	Fomu 02<br>**Ukurasa la 1 ya 17**

**Nambari ya Kituo #**<br>_ _ _ _	**Nambari ya Kushiriki #**<br>_ _ _ _ _ _	**Nambari ya Kuzuru#**<br>_ _	**Tarehe ya Kuzuru**<br>_ _ / _ _ / _ _ _ _<br>dd mm y y y y	**Kitambulishi cha Anayehoji**<br>_ _

Maelezo: Jaza Orodha ya Maswali kuhusu mienendo Fomu 02 wakati wa kusajili, mwezi wa 6, mwezi wa 12, mwezi wa 18, na mwezi wa 24 wa kurudi kwenye utafiti. DK = sijui, RE = amekataa kujibu.

“Tafadhali kumbuka kuwa si lazima ujibu maswali yoyote ambayo hupendi kuyajibu na waweza kutamatisha maojiano wakati wowote.”

Sehemu 1: Habari kumuhusu mshiriki

1. Je, umeajiriwa wakati huu? **1 = Ndiyo** **2 = La** **28 = DK** **29 = RE**

Kama ndiyo,unafanya nini wakati huu kujimudu kimaisha:

1= Nimeajiriwa kwa kudumu
2 = Nimeajiriwa kwa njia ya kibarua, kwa mkataba mfupi, au kwa hali ya kila siku
3 = Nimejiajiri

Kama La:

4 = Sijajiriwa na natafuta kazi
5 = Mtunza boma bila kazi ingine nje
6 = Mwanafunzi
7 = Nimestahafu au kilema
8 = Ingine (eleza): ______________________

2. Mapato yako kwa mwezi ukikadiria ni kiasi gani kwa miezi 12 iliyopita ? ___________

3. Je, una umeme kwa nyumba yako? 1 = Ndiyo 2 = La

4. Ni watu wangapi wanaishi nawe kwa nyumba yako?__________

5. Ni watu wangapi wanatumia kwa pamoja chumba chako cha kuogea?__________

6. Maji ya kutumia yako karibu na nyumba yako vipi?
1 = Ndani ya nyumba (maji ya mfereji)
2 = Nje tu ya nyumba (kwa eneo lako)
3 = Chini ya dakika 10 ukitembea kutoka kwa nyumba yako
4 = Zaidi ya dakika 10 ukitembea kutoka kwa nyumba yako

7. Unaishi na nani kwa wakati huu?
1 = Peke yangu
2 = Mke mangu/mshirika wa kike niayeishi naye
3 = Familia
4 = Rafiki(ma)
5 = Ingine _____________________
28 = DK
29 = RE

8. Je, hali yako ya ndoa ni ipi?

1 = Sijaoa, bila mshirika wa kuishi naye →(Nenda kwa 11)
2 = Sijaoa, na mshirika wa kuishi naye
3 = Nimeoa, naishi na bibi yangu
4 = Nimeoa siishi na bibi yangu
28 = DK
29 = RE

9. Una wake /washirika wangapi?

Wake / washirika _________

10. Usiku uliopita je, wewe mke au mshirika wako unayeishi naye mlilala kwa nyumba moja?

1 = Ndiyo 2 = La 28 = DK 29 = RE

11. Kwa miezi 6 zilizopita, ni safari ngapi zaidi ya usiku mmoja uliweza kusafiri?

__ __ __

Sehemu 2: Uwezekano wa Kujiusisha na damu

12. Je, umewai kutoa damu?

1 = Ndiyo 2 = La 28 = DK 29 = RE

12a. kama ndiyo, mara ngapi kwa miezi 6 zilizopita?

____

13. Je, umewai kuongezwa damu hosipitalini?

1 = Ndiyo 2 = La 28 = DK 29 = RE

13a. Kama ndiyo, mara ngapi kwa miezi 6 zilizopita?

____

14. Je, umewai kuguswa na damu ya mtu yeyote?

1 = Ndiyo 2 = La 28 = DK 29 = RE

14a. Kama ndiyo, mara ngapi kwa miezi 6 zilizopita?

____

14b. Tafadhali eleza kwa ufupi ni kitu gain kilitendeka (ajali, moto, na kadhalika.)

____

15. Je, umewai kuchorwa ngozi?

1 = Ndiyo 2 = La 28 = DK 29 = RE

15a. Kama ndiyo, mara ngapi kwa miezi 6 zilizopita?

____

16. Je, umewai kuchanjwa ngozi kwa sababu zozote za kiafya au zinginezo?

1 = Ndiyo 2 = La 28 = DK 29 = RE

16a. Kama ndiyo, mara ngapi kwa miezi 6 zilizopita?

____

17. Je, umewai kudungwa sindano kwa sababu zozote?

1 = Ndiyo 2 = La 28 = DK 29 = RE

17a. Kama ndiyo, mara ngapi kwa miezi 6 zilizopita?

____

17b. Kama ndiyo, ni mara ngapi zilikuwa kwa matibabu ya ugonjwa wa zinaa kwa miezi 6 zilizopita?

____

18. Katika miezi 6 zilizopita, je umewai kudungwa na sindano au kukatwa na kisu kwa sababu zozote? 1 = Ndiyo 2 = La 28 = DK 29 = RE

18a. Kama ndiyo, mara ngapi? ________________________________

18b. Kama ndiyo, ni nini baadhi ya sababu hizo? ________________________________

Sehemu 3: Utendaji wa ngono

Sasa nitakuuliza maswali nyeti. Yanahusu kuhusika kwako na utendaji wa ngono. Kumbuka kama utaahibika au utaki kujibu swali lolote, waweza kukataa kujibu. Lakini, ukikubali kujibu swali, tafadhali uwe mwaminifu.

19. Je, umewai kushiriki ngono ya uke na msichana/mwanamke? 1 = Ndiyo 2 = La 29 = RE (kama la, enda kwa 34)

20. Ulikuwa na umri gani(miaka) ulipofanya ngono na msichana/mwanamke kwa mara ya kwanza? __ __ miaka

21. Wasichana/wanawake wa ngapi tofauti ikiwemo mke/wake wako umeshiriki nao ngono…..

21a. Maishani mwako? __ __ __

21b. Miezi 12 zilizopita? __ __ __

21c. Miezi 6 zilizopita? __ __ __

21d. Siku 30 zilizopita? __ __ __

22. Je, umeshiriki ngono kwa miezi 6 zilizopita? 1 = Ndiyo 2 = La 29 = RE

23. Ni muda mrefu kiasi gani tangu ushiriki ngono??

__ __ siku

__ __ majuma

__ __ miezi

__ __ miaka

24. Ni mara ngapi umeshiriki ngono ikiwa ni pamoja na mke wako na mshirika yeyote kwa:

24a. Siku 7 zilizopita __ __

24b. Siku 30 zilizopita __ __

25. Kwa miezi 6 zilizopita, je umewai:

25a. Ulishiriki ngono katika hali ambayo pesa au zawadi zilibadilishwa? 1 = Ndiyo 2 = La 29 = RE

25b. Ulishiriki ngono na mwanamke kama anahedhi? 1 = Ndiyo 2 = La 29 = RE

25c. Ulishiriki ngono na washirika 2 au washirika zaid kwa muda huo wa siku 30? 1 = Ndiyo 2 = La 29 = RE

26. Je, umewaikushiriki ngono na washirika wawili au zaidi kwa muda wa siku 30? 1 = Ndiyo 2 = La 29 = RE

27. Mara ya mwisho uliposhiriki ngono..

27a. Umekua ukikunywa pombe? 1 = Ndiyo 2 = La 29 = RE

27b. Je, ulitumia mpira wa "kondomu"? 1 = Ndiyo 2 = La 29 = RE

27c. Ilikuwa ni na… 1 = mke wako 2 = mshirika wa kawaida 3 = mshirika asiye wa kawaida 4 = mshirika ngono wa kulipwa 28 = DK 29 = RE

28. Ni mara ngapi umeuweka ulimi wako au kulamba uke wa mshirika wako? 1 = Kamwe 2 = si mara nyingi 3 = wakati mwingine 4 = Mara nyingi 5 = Kila mara 28 = DK 29 = RE

29. Ni mara ngapi umeuingiza uume wako kwenye mkundu wa mshirika wako? 1 = Kamwe 2 = si mara nyingi 3 = wakati mwingine 4 = Mara nyingi 5 = Kila mara 28 = DK 29 = RE

30. Ni mara ngapi umeshiriki ngono na mwanamke siku hiyo ulikutana naye? 1 = Kamwe 2 = si mara nyingi 3 = wakati mwingine 4 = Mara nyingi 5 = Kila mara 28 = DK 29 = RE

31. Ni mara ngapi umeshiriki ngono baada ya kupaka kitu chochote(dawa ya kienyeji,mafuta ya mwili na vilainisho, na kadhalika) kwenye uume wako? 1 = Kamwe 2 = si mara nyingi 3 = wakati mwingine 4 = Mara nyingi 5 = Kila mara 28 = DK 29 = RE

31a. Kama umewai, ni vitu gani? ____

32. Ni mara ngapi umeshiriki ngono baada ya mshirika wako kupaka kitu chochote kwenye uke au ndani ya uke wake? 1 = Kamwe 2 = si mara nyingi 3 = wakati mwingine 4 = Mara nyingi 5 = Kila mara 28 = DK 29 = RE

32a. Kama umewai, ni vitu gani? ____

33. Je, ungependelea kushiriki ngono kama uke wa mshirika wako…….(soma majibu)
1 = Umekauka wakati wa ngono
2 = Haujakauka wakati wa ngono
3 = Hauna pendekezo lolote

Kama imekauka, kwa ujuzi wako, jambo gani mwanamke ulifanya kuukausha uke wake?

33a. Anapanguza uke wake na taulo/nguo 1 = Ndiyo 2 = La 28 = DK 29 = RE

33b. Anatumia madawa ya kienyeji au poda 1 = Ndiyo 2 = La 28 = DK 29 = RE

33c. Anatumia bidhaa vya biashara 11 = Ndiyo 2 = La 28 = DK 29 = RE

vinavyopatikana (kwa mfano, madawa ya kuuwa viini, sabuni, na kadhalika)

**34. Je, umewai kushiriki ngono na mvulana au msichana?**	1 = Ndiyo 2 = La 28 = DK 29 = RE<br><br>La →(enda kwa 39)
**35. kama ndiyo, wavulana/wanaume ngapi?**	__ __
**36. je umewai kuuingiza uume wako kwenye mkundu wa mwanaume mwingine?**	1 = Ndiyo 2 = La 29 = RE
**37. Je, uume umewai kuingizwa kwenye mkundu wako?**	1 = Ndiyo 2 = La 29 = RE
**38. Je mwanaume amewai kumwaga shahawa kwenye mdomo wako?**	1 = Ndiyo 2 = La 29 = RE

Sehemu 4: Pombe na Utumizi wa Madawa ya kulevya

**39. Je, ulikunywa mvinyo iliyo na pombe kama bia, spirits, changaa, busaa na kadhalika kwa majuma manne yaliyopita?**	1 = Yes 2 = No 28 = DK 29 = RE<br>LA →(Enda kwa 42)
**40. Ni mara ngapi ulikunywa mvinyo zilizo na pombe kwa majuma manne yaliyopita. Waweza ukasema…….?**	1 = Angalau mara moja kwa siku<br>2 = Angalau mara moja kwa juma<br>3 = Kidogo ya mara moja kwa juma
**41. Kwa miezi 6 zilizopita, ni mara ngapi umeshiriki ngono baada ya au ukiwa umekunywa pombe?**	1 = Kamwe 2 = Wakati mwingine 3 = Mara nyingi<br>4 = Kila mara 28 = DK 29 = RE
**42. Watu wengine wamejaribu kutumia baadhi ya madawa tofauti ya kulevya. Ni gani yoyote kati ya haya, umeijaribu?**	
**42a. Bhangi / njaga**	1 = Ndiyo 2 = La
**42b. Mandrax**	1 = Ndiyo 2 = La
**42c. Valium**	1 = Ndiyo 2 = La
**42d. Glue**	1 = Ndiyo 2 = La
**42e. Miraa**	1 = Ndiyo 2 = La
**42f. Kuber**	1 = Ndiyo 2 = La
**42g. Ingine (Eleza)**	______________________________
**43. Watu wengine wamejaribu kujidunga madawa wakitumia sindano. Je, umewai kujidunga madawa (si kwa sababu ya tiba?)**	1 = Ndiyo 2 = La 28 = DK 29 = RE
**44. Kwa miezi 6 zilizopita, ni mara ngapi umeshiriki ngono kama umetumia au baada ya kutumia baadhi**	1 = Kamwe 2 = Wakati mwingine 3 = Mara nyingi

**ya madawa haya?**	4 = Kila mara 28 = DK 29 = RE

Sehemu 5: Utumizi wa kondomu

**45. Je, umewai kutumia kondomu?**	1 = Ndiyo 2 = La 28 = DK 29 = RE<br>La →(Enda kwa 49)
**46. Je, umewai kuwa na shida ukitumia kondomu? Kama ndiyo, shida aina gani:**	1 = Ndiyo 2 = La 28 = DK 29 = RE
**46a. Sikujua jinsi ya kutumia kondomu**	1 = Ndiyo 2 = La
**46b. Kondomu ilipasuka**	1 = Ndiyo 2 = La
**46c. Kondomu iliteleza na kutoka wakati wa ngono**	1 = Ndiyo 2 = La
**46d. Kondomu ilikuwa pana sana**	1 = Ndiyo 2 = La
**46e. Kondomu ilikuwa ndogo sana**	1 = Ndiyo 2 = La
**46f. Ingine (Eleza)**	________________________
**47. Kwa miezi 6 zilizopita, wafikiri imekuwa raisi au vigumu kwako kupata kondomu?**	1 = Raisi sana 2 = Raisi kidogo<br>3 = Vigumu kidogo 4 = Vigumu sana<br>28 = DK 29 = RE
**48. Je, kwa wakati huu una kondomu?**	1 = Ndiyo 2 = La 29 = RE

Sehemu 6: Historia ya afya ya Uzazi

“Sasa nitakuuliza maswali mengine kuhusu afya yako ya uzazi. Wanaume wengine husikia maumivu wakikojowa, wanatoa usaa kwenye uume, au wanavidonda kwenye uume wao.”

49. Kwa miezi sita zilizopita, umewai kuwa na:

**49a. Maumivu ukikojoa**	1 = Ndiyo 2 = La
**49b. Kukojoa mara nyingi**	1 = Ndiyo 2 = La
**49c. Vidonda kwenye uume**	1 = Ndiyo 2 = La
**49d. Toa usaa kwenye uume**	1 = Ndiyo 2 = La
**49e. Ugumu kukojoa (Inabidi unakawia muda mrefu ili mikojo itoke)**	1 = Ndiyo 2 = La
**49f. Maumivu ukishiriki ngono**	1 = Ndiyo 2 = La
**49g. Kuvuja damu ukishiriki ngono**	1 = Ndiyo 2 = La
**49h. Maumivu chini ya tumbo**	1 = Ndiyo 2 = La
**49i. Vitu vimemea kwenye uume wako**	1 = Ndiyo 2 = La
**50. Kama ndiyo kwa lolote kwenye nambari 49: Je, ulishiriki ngono ukiwa na shida hizi?**	1 = Ndiyo 2 = La 28 = DK 29 = RE

**51. Je, daktari au wauguzi wengine WAMEWAI kukuambia kwamba ulikuwa na ugonjwa wa zinaa?**	1 = Ndiyo 2 = La 28 = DK 29 = RE
**51a. Kama ndiyo, je wakumbuka ilikuwa ni ugonjwa gani wa zinaa?**	______________________________
**52. Je, ulikuwa na ugonjwa wa zinaa kwa miezi 6 zilizopita?**	1 = Ndiyo 2 = La 28 = DK 29 = RE
**53. Je, UMEWAI kupata matibabu ya ugonjwa wa zinaa?**	1 = Ndiyo 2 = La 28 = DK 29 = RE
**53a. Kama ndiyo, mara ngapi?**	____________
**53b. Ulidungwa sindano kwa matibabu?**	1 = Ndiyo 2 = La
**53c. Je, ulitibiwa kwa ajili ya ugonjwa wa zinaa kwa miezi 6 iliyopita?**	1 = Ndiyo 2 = La

Sehemu 7: Utendaji ngono na Kuridhika

**54. Kwa miezi 6 zilizopita, je kumewai kuwepo na muda wa majuma mawili au zaidi ambapo uli……..**	
**54a. Hukuwa na haja ya kushiriki ngono?**	1 = Ndiyo 2 = La 28 = DK 29 = RE
**54b. Hungeweza kufikia kilele cha ngono(weza kumwaga shahawa)?**	1 = Ndiyo 2 = La 28 = DK 29 = RE
**54c. Ulifikia kilele cha ngono (weza kumwaga shahawa) kwa haraka?**	1 = Ndiyo 2 = La 28 = DK 29 = RE
**54d. Ulikuwa na maumivu ukishiriki ngono?**	1 = Ndiyo 2 = La 28 = DK 29 = RE
**54e. Hukupata ngono kuwa ya kuburudisha (hata kama hakukuwa na maumivu)?**	1 = Ndiyo 2 = La 28 = DK 29 = RE
**54f. Ulikuwa na shida ya kutuna au kuudumisha uume wako wima?**	1 = Ndiyo 2 = La 28 = DK 29 = RE
**55. Kwa miezi 6 zilizopita,ni mara ngapi ukishiriki ngono uume wako ulikuuma?**	1 = Kamwe 2 = Si mara nyingi 3 = Wakati mwingine<br>4 = Mara nyingi 5 = Kila mara 28 = DK 29 = RE
**56. Kwa miezi 6 zilizopita,ni mara ngapi ukishiriki ngono ambapo ngozi ya uume wako ulikwaruzwa, ulikatika, au ulichubuka?**	1 = Kamwe 2 = Si mara nyingi 3 = Wakati mwingine<br>4 = Mara nyingi 5 = Kila mara 28 = DK 29 = RE
**57. Kwa miezi 6 zilizopita,ni mara ngapi ukishiriki ngono au baada ya ngono ambapo ngozi ya uume wako ilivuja damu?**	1 = Kamwe 2 = Si mara nyingi 3 = Wakati mwingine<br>4 = Mara nyingi 5 = Kila mara 28 = DK 29 = RE
**58. Kwa zaidi ya miezi 6 zilizopita, je kwa ujumla utaweza kukisia kuridhishwa kwako na ngono namna gani?**	1 = Sijaridhika hata kidogo 2 = Sijaridhika<br>3 = Ridhika 4 = Ridhika sana<br>28 = DK 29 = RE

Tukiangaza zaidi, je umeridhishwa namna gani na…..

**58a. Kiwango cha hamu kushiriki ngono**	1 = Sijaridhika hata kidogo	2 = Sijaridhika
	3 = Ridhika	4 = Ridhika sana
	28 = DK	29 = RE
**58b. Uume kutuna**	1 = Sijaridhika hata kidogo	2 = Sijaridhika
	3 = Ridhika	4 = Ridhika sana
	28 = DK	29 = RE
**58c. Kudumisha kutuna**	1 = Sijaridhika hata kidogo	2 = Sijaridhika
	3 = Ridhika	4 = Ridhika sana
	28 = DK	29 = RE
**58d.Uwezekano wa kuuingiza uume wako ndani ya uke wa mshirika wako**	1 = Sijaridhika hata kidogo	2 = Sijaridhika
	3 = Ridhika	4 = Ridhika sana
	28 = DK	29 = RE
**58e. Nafasi kati ya kila kutuna**	1 = Sijaridhika hata kidogo	2 = Sijaridhika
	3 = Ridhika	4 = Ridhika sana
	28 = DK	29 = RE
**58f. Uwezekano wa kumwaga shahawa**	1 = Sijaridhika hata kidogo	2 = Sijaridhika
	3 = Ridhika	4 = Ridhika sana
	28 = DK	29 = RE
**58g. Kiwango cha maumivu wakati wa ngono**	1 = Sijaridhika hata kidogo	2 = Sijaridhika
	3 = Ridhika	4 = Ridhika sana
	28 = DK	29 = RE
**58h. Maoni ya mchumba/Mshirika kuhusu jinsi ninavyoshiriki ngono**	1 = Sijaridhika hata kidogo	2 = Sijaridhika
	3 = Ridhika	4 = Ridhika sana
	28 = DK	29 = RE

Sehemu 8: Maono kuhusu hatari na utendaji wa ngono

59. Kwa miezi 6 zilizopita, je unaamini utendaji wako ngono ume:

1 = Umepungua →Enda kwa 60
2 = Umeongezeka →Enda kwa 61
3 = Umebaki jinsi ilivyokuw →Enda kwa 62
28 = DK →Enda kwa 62
29 = RE →Enda kwa 62

60. Ni baadhi ya sababu gani utendaji ngono wako umepunguka kwa miezi 6zilizopita?

**60a. Uoga kuambukizwa viini vya UKIMWI**	1 = Ndiyo 2 = La
**60b. Nafasi chache za kushiriki ngono**	1 = Ndiyo 2 = La
**60c. Ushawishi kotoka kwa vyombo vya habari, jamii, marafiki, na kadhalika.**	1 = Ndiyo 2 = La
**60d. Uamuzi wangu kupunguza utendaji ngono**	1 = Ndiyo 2 = La
**60e. Ingine (eleza)**	______________________

61. Ni baadhi ya sababu gani utendaji ngono wako umeongezeka kwa miezi 6 zilizopita?

**61a. Kuongezeka kwa nyege(tamaa ya ngono)**	1 = Ndiyo 2 = La
**61b. Nafasi nyingi za kushiriki ngono(washirika wamekuwa wengi na wanataka)**	1 = Ndiyo 2 = La
**61c. Ushawishi kotoka kwa vyombo vya habari, jamii, marafiki, na kadhalika.**	1 = Ndiyo 2 = La
**61d. Tohara**	1 = Ndiyo 2 = La
**61e. Ingine (eleza)**	______________________

62. Unafikiri nafasi yako kuambukizwa viini vya UKIMWI ni kiasi gani?

1 = Hakuna nafasi kamwe
2 = Nafasi kidogo
3 = Nafasi kutosha →Enda kwa 64
4 = nafasi kubwa →Enda kwa 64
5 = Sijui →Enda kwa 64

63. Kwa nini unafikiri hauna nafasi kamwe au nafasi kidogo kuambukizwa viini vya UKIMWI?

Ikijibiwa, enda kwa 65

**63a. Si shiriki ngono**	1 = Ndiyo 2 = La
**63b. Natumia kondomu kila mara**	1 = Ndiyo 2 = La
**63c. Nina mshirika mmoja pekee**	1 = Ndiyo 2 = La

63d. Nimepunuza idadi ya washirika 1 = Ndiyo 2 = La

63e. Mshirika ni mwaminifu kwangu 1 = Ndiyo 2 = La

63f. Tohara 1 = Ndiyo 2 = La

63g. Ingine (eleza) ____

64. Kwa nini unafikiri una nafasi kutosha au nafasi kubwa kuambukizwa viini vya UKIMWI?

64a. Situmii kondomu 1 = Ndiyo 2 = La

64b. Nina washirika ngono zaidi ya mmoja 1 = Ndiyo 2 = La

64c. Mshirika ana washirika wengine 1 = Ndiyo 2 = La

64d. Inmehusika na washoga 1 = Ndiyo 2 = La

64e. Niliongezwa damu/ sindano 1 = Ndiyo 2 = La

64f. Nilipashwa tohara 1 = Ndiyo 2 = La

64g. Ingine (eleza) ____________________________________

65. Ni kitendo kipi, kwa maoni yako, inamweka mtu hatarini kubwa kuambukizwa viini vya UKIMWI?

65a. Ngono kwenye uke au mkundu?

1 = Ngono bila kinga kwenye uke ni hatari kubwa
2 = Ngono bila kinga kwenye mkundu ni hatari kubwa
3 = Zote zina hatari sawa kwa viini vya UKIMWI
28 = DK 29 = RE

65a. Kavu (kama mshirika wako ame ukausha uke wake na nguo au kitu) au ngono isiyokavu?

1 = Ngono bila kinga isiyokavu ni hatari kubwa
2 = Ngono bila kinga iliyokavu ni hatari kubwa
3 = Zote zina hatari sawa kwa viini vya UKIMWI
28 = DK 29 = RE

66. Je, unakubaliana na maneno haya?

66a. Inachukua bidii nyingi ili kuifanya mienendo yako ya ngono kuwa salama. 1 = Nakubali 2 = Sina uhakika 3 = Sikubali

66b. Unachoshwa na kuchunguza mienendo yako ya ngono kila mara. 1 = Nakubali 2 = Sina uhakika 3 = Sikubali

66c. Ukiwa juu au umelewa, kuna uwezekano mwingi kushiriki ngono na watu mbali na mshirika wako wa kawaida. 1 = Nakubali 2 = Sina uhakika 3 = Sikubali

66d. Ukiwa juu au umelewa, kuna uwezekano mwingi kushiriki ngono bila kutumia kondomu. 1 = Nakubali 2 = Sina uhakika 3 = Sikubali

Sehemu 9: Itikadi kuhusu ngono na mambo ya ngono.

67. Tafadhali niambie kama unakubaliana au haukubaliani na maneno yafuatayo.

**67a. Wanaume wanaweza kufurahia ngono hata na washirika ambao hawapendi**	1 = Nakubali zaidi 2 = Nakubali 3 = Sina uhakika 4 = Sikubali 5 = Sikubali zaidi 29 = RE
**67b. Mwanaume "kamili" yuko tayari kwa ngono wakati wowote**	1 = Nakubali zaidi 2 = Nakubali 3 = Sina uhakika 4 = Sikubali 5 = Sikubali zaidi 29 = RE
**67c. Wanawake wanauwezo mkubwa kudhibiti tamaa ya ngono kuliko wanaume**	1 = Nakubali zaidi 2 = Nakubali 3 = Sina uhakika 4 = Sikubali 5 = Sikubali zaidi 29 = RE
**67d. Ngono ni sehemu muhimu kwa maisha**	1 = Nakubali zaidi 2 = Nakubali 3 = Sina uhakika 4 = Sikubali 5 = Sikubali zaidi 29 = RE
**67e. Kutokuwa na uwezo wa kushiriki ngono ni chanzo cha uzuni kwa washirika**	1 = Nakubali zaidi 2 = Nakubali 3 = Sina uhakika 4 = Sikubali 5 = Sikubali zaidi 29 = RE
**67f. Ubora wa uume (kutuna) kusimama wima ndiyo inaridhisha mwanamke**	1 = Nakubali zaidi 2 = Nakubali 3 = Sina uhakika 4 = Sikubali 5 = Sikubali zaidi 29 = RE
**67g. Mwanaume kamili anashiriki ngono maranyingi**	1 = Nakubali zaidi 2 = Nakubali 3 = Sina uhakika 4 = Sikubali 5 = Sikubali zaidi 29 = RE
**67h. Wanawake ambao hawavutii katika hali ya ngono hawawezi kuridhika na ngono**	1 = Nakubali zaidi 2 = Nakubali 3 = Sina uhakika 4 = Sikubali 5 = Sikubali zaidi 29 = RE
**67i. Ninafurahia mwili wangu jinsi ilivyo**	1 = Nakubali zaidi 2 = Nakubali 3 = Sina uhakika 4 = Sikubali 5 = Sikubali zaidi 29 = RE

Sehemu 10: Itikadi kuhusu tohara

**68. Ni raisi kuweka uume uwe safi kama….?**	1 = Umepashwa tohara 2 = Hujapashwa tohara 3 = Hakuna tofauti 28 = DK 29 = RE
**69. Ni raisi kupata ugonjwa kutoka kwa mwanamke kama …?**	1 = Umepashwa tohara 2 = Hujapashwa tohara 3 = Hakuna tofauti 28 = DK 29 = RE
**70. Ni raisi kupata UKIMWI kama …?**	1 = Umepashwa tohara 2 = Hujapashwa tohara 3 = Hakuna tofauti 28 = DK 29 = RE
**71. Wanaume hupata ngono ni ya kuburudisha mno kama …?**	1 = Umepashwa tohara 2 = Hujapashwa tohara 3 = Hakuna tofauti 28 = DK 29 = RE

72. Wengi wa wanawake hupata ngono ni ya kuburudisha mno wakishiki ngono na wanaume ambao….

1 = Umepashwa tohara 2 = Hujapashwa tohara
3 = Hakuna tofauti 28 = DK 29 = RE

73. Wanaume ambao….. wanashiriki ngono kiolela

1 = Umepashwa tohara 2 = Hujapashwa tohara
3 = Hakuna tofauti 28 = DK 29 = RE

74. Tafadhali toa maoni yako kuhusu tohara:

74a. Sasa vile tohara inapatikana, viini vya UKIMWI si tisho sana jinsi ilivyokuwa mbeleni.

1 = Nakubali 2 = Sina uhakika 3 = Sikubali

74b. Sasa vile tohara inapatikana, utumizi wa kondomu wakati wa ngono si muhimu vile.

1 = Nakubali 2 = Sina uhakika 3 = Sikubali

74c. Sasa vile tohara inapatikana, hauna hofu sana kuhusu viini vya UKIMWI.

1 = Nakubali 2 = Sina uhakika 3 = Sikubali

74d. Sasa vile tohara inapatikana, kunauwezekano kwako kuwa na washirika wa ngono zaidi ya mmoja.

1 = Nakubali 2 = Sina uhakika 3 = Sikubali

74e. Sasa vile tohara inapatikana, waweza kuchukua fursa ya kuambukizwa au kumuambukiza mwingine viini vya UKIMWI.

1 = Nakubali 2 = Sina uhakika 3 = Sikubali

74f. Sasa vile tohara inapatikana, mtu ambaye ameambukizwa viini vya UKIMWI haitajiki kuwa na hofu sana kuhusu utumizi wa kondomu.

1 = Nakubali 2 = Sina uhakika 3 = Sikubali

74g. Sasa vile tohara inapatikana, kuna uwezekano kwako kushiriki ngono bila kondomu.

1 = Nakubali 2 = Sina uhakika 3 = Sikubali

Sehemu 11: Usafi

75. Ni mara ngapi umekua ukiosha uume wako?

1 = Mara moja kwa mwezi au chini
2 = Mara nyingi kwa mwezi
3 = mara moja kwa juma
4 = Mara nyingi kwa wiki
5 = Kila siku
6 = Sioshi uume wangu

76. Mara ya mwisho uliposhiriki ngono, je uliosha uume wako punde tu baada ya ngono?

1 = Ndiyo 2 = La 28 = DK 29 = RE

Nini ulitumia kupanguza uume wako?

76a. Nguo/kijitambaa bila maji 1 = Ndiyo 2 = La 28 = DK 29 = RE

76b. Maji 1 = Ndiyo 2 = La 28 = DK 29 = RE

76c. Sabuni na maji 1 = Ndiyo 2 = La 28 = DK 29 = RE

76d. Madawa ya kienyeji 1 = Ndiyo 2 = La 28 = DK 29 = RE

76e. Ingine (eleza) ________________________________

76f. Kama ndiyo, ilichukua muda upi ili uioshe uume wako baada ya ngono? _______masaa _____ dakika

77. Je, umewai kuweka au kupaka vitu fulani kwa sehemu ya juu ya uume wako kwa sababu zozote?
1 = Ndiyo
2 = La 28 = DK 29 = RE →enda kwa 78

77a. Kama ndiyo, ni sababu gani ulipaka vitu hivyo?
1 = Kwa kusafisha/kuua viini
2 = Kutoa harufu mbaya
3 = Kuzuia mimba
4 = Kuponya kuwashwa
5 = Baada ya ngono kutakasa
6 = Kabla ya ngono kuanzisha/kujitayarisha
7 = Kuponya vidonda
8 = Kujikinga dhidi ya ugonjwa/viini
9 = Kumkinga mshirika dhidi ya ugonjwa/viini
29 = RE

Sehemu 12: Kupimwa viini vya UKIMWI na Kushauriwa

78. Je, umewai kupimwa kama una viini vya UKIMWI? 1 = Ndiyo 2 = La 28 = DK 29 = RE

79. Ulipopimwa viini vya UKIMWI, je ulipata habari jinsi unaweza kupunguza hatari ya kuambukizwa viini vya UKIMWI? 1 = Ndiyo 2 = La 28 = DK 29 = RE

80. Ni mara ngapi umepimwa viini vya UKIMWI? __ __

81. Ulipimwa lini mwisho?
1 = Chini ya mwaka moja uliopita
2 = Mwaka 1-2 iliyopita
3 = Zaidi ya miaka 2 iliyopita

82. Nitakuuliza kuhusu matokeo yako ya kupimwa viini, lakini waweza kukataa kujibu ukipenda. Matokeo yako yamwisho kuhusu viini vya UKIMWI yalikuwa gani?
1 = Viini vya UKIMWI vilipatikana
2 = Viini vya UKIMWI havikupatikana →enda kwa 85
3 = Sikupata matokeo →enda kwa 85
29 = RE →enda kwa 85

83. Je waenda kliniki kupokea huduma kwa ajili ya Viini vya UKIMWI? 1 = Ndiyo 2 = La 28 = DK 29 = RE

84. Je, unapokea matibabu ya kukabiliana na makali ya viini vya UKIMWI (ART)? 1 = Ndiyo 2 = La 28 = DK 29 = RE

Sehemu 13: Tohara

85. Je, umepashwa tohara?
1 = Ndiyo
2 = La → Enda kwa 102

86. Ulipashwa wapi tohara?
1 = Kwenye kliniki hii
2 = Ingine (eleza)______________________

87. Ni muda kiasi gani tangu upashwe tohara?
__ __ Siku
__ __ Majuma
__ __ Miezi

88. Umeridhika kiasi gani ….

88a.Jinsi tohara yako ilivyofanywa?
1 = Ridhika zaidi 2 = Ridhika ya kutosha
3 = Sina maoni 4 = Sija ridhika vile
5 = Sija ridhika hata kidogo 28 = DK 29 = RE

88b. Jinsi uume wako unavyofanana?
1 = Ridhika zaidi 2 = Ridhika ya kutosha
3 = Sina maoni 4 = Sija ridhika vile
5 = Sija ridhika hata kidogo 28 = DK 29 = RE

88c. Jinsi unavyoshiriki ngono?
1 = Ridhika zaidi 2 = Ridhika ya kutosha
3 = Sina maoni 4 = Sija ridhika vile
5 = Sija ridhika hata kidogo 28 = DK 29 = RE

89. Je, umewai kutuna tangu upashwe tohara? 1 = Ndiyo 2 = La 29 = RE

89a. Kama la, kwa nini?

Kuna uma 1 = Ndiyo 2 = La 29 = RE

Kuna vuja damu 1 = Ndiyo 2 = La 29 = RE

Kuna bana/kunashika sana 1 = Ndiyo 2 = La 29 = RE

89b. Kama ndiyo kwa 90, je kutuna kwako ni wa kawaida? 1 = Ndiyo 2 = La 29 = RE

90. Mbali na ulipokua ukipona, je umewai kujiepusha na ngono kwa sababu umepashwa tohara? 1 = Ndiyo 2 = La 29 = RE

91. Je mmeshiriki ngono tangu upashwe tohara?

1 = Ndiyo
2 = La 29 = RE → Enda kwa 97f

92. Ni karibu muda gani baada ya tohara ambapo ulishiriki ngono kwa mara ya kwanza?

__ __ Saa
__ __ Siku
__ __ Wiki
__ __ Miezi

93. Je, umetumia kondomu tangu upashwe tohara?

1 = Ndiyo
2 = La 29 = RE → Enda kwa 95

94. Je, uligundua kuwa ni raisi kutumia kondomu baada ya tohara, ukilinganisha na kabla upashwe tohara?

1 =Raisi baada ya tohara
2 =Raisi kabla ya tohara
3 =Karibu sawa
4 = Sikuwa nimetumia kondomu kabla ya tohara
28=DK
29=RE

95. Je, uligundua kwamba ngono ni ya kuburudisha mno kabla upashwe tohara au baada ya kupashwa tohara?

1 = Ya kuburudisha mno kabla ya tohara
2 = Ya kuburudisha mno baada ya tohara
3 = Hakuna tofauti
28 = DK
29 = RE

96. Je wafikiri kwamba ngono ilikuwa ya kuburudisha mno kwa washirika wako kabla ya tohara au baada ya tohara?

1 = Ya kuburudisha mno kabla ya tohara
2 = Ya kuburudisha mno baada ya tohara
3 = Hakuna tofauti
28 = DK
29 = RE

97. Ukilinganisha na kabla upashwe tohara:

97a. Unaweza kusema uume wako unajihisi kiasi gani?

1=Zaidi kidogo 2=Kiasi kutosha
3=Karibu sawa 4=Kidogo
5=Kidogo sana 28=DK 29=RE

97b. Je, wadhani unafikia kilele cha ngono kwa uraisi kiasi gani?

1=Zaidi kidogo 2=Kiasi kutosha
3=Karibu sawa 4=Kidogo
5=Kidogo sana 28=DK 29=RE

97c. Je, umekuwa ukishiriki ngono kwa wingi kiasi gani?

1=Zaidi kidogo 2=Kiasi kutosha

	3=Karibu sawa 4=Kidogo<br>5=Kidogo sana 28=DK 29=RE
**97d. Je, una shida zaidi, kidogo au sawa ya kuchubuka,kupasuka au kukatika ngozi?**	1=Zaidi kidogo 2=Kiasi kutosha<br>3=Karibu sawa 4=Kidogo<br>5=Kidogo sana 6= Sikuwa nachubuka mbeleni<br>28=DK 29=RE
**97e. Je, unamwaga shahawa mapema kuliko vile ulipendelea?**	1=Mapema zaidi 2 =Kiasi kutosha<br>3=Karibu sawa 4=Kidogo<br>5=Kidogo sana 6= Sikumwaka shahawa mapema hapo mbeleni<br>28=DK 29=RE
**97f. Je, wadhani unakinga dhidi ya viini vya UKIMWI kiasi gani?**	1=Zaidi kidogo 2=Kiasi kutosha<br>3=Karibu sawa 4=Kidogo<br>5=Kidogo sana 28=DK 29=RE
**97g. Je, kulenga kwako wakati wa kukojoa bora, mbaya, au karibu ni sawa?**	1=Bora zaidi 2=Kiasi kutosha<br>3=Karibu sawa 4=mbaya kidogo<br>5=Mbaya sana 28=DK 29=RE
**98. Je, tohara imebadili tabia yako ya ngono kwa njia yoyote?**	1 = Ndiyo 2 = La 28 = DK 29 = RE
**99. Tangu utahiriwe, unavutia wanawake kiasi gani?**	1=Zaidi kidogo 2=Kiasi kutosha<br>3=Karibu sawa 4=Kidogo<br>5=Kidogo sana 28=DK 29=RE
**100. Isipokuwa washirika wako, kuna mtu yeyote katika familia yetu anajua ya umepashwa tohara?**	1 = Ndiyo 2 = La 28 = DK 29 = RE
**100a. Kama ndiyo, kwa ujumla, maoni yao ni gani?**	1=Walipendezwa sana<br>2=Walipendezwa kiasi<br>3=Hawakuonyesha lolote/hawakutoa maoni yoyote<br>4=Hawakupendezwa vile<br>5=Hawakupendezwa hata kidogo<br>28=DK 29=RE
**101. Je, washirika wako wowote wanajua umepashwa tohara?**	1 = Ndiyo 2 = La 28 = DK 29 = RE
**102a. kama ndiyo, walipendezwa au**	1=Walipendezwa sana

**hawakupendezwa?**	2=Walipendezwa kiasi<br>3=Hawakuonyesha lolote/hawakutoa maoni yoyote<br>4=Hawakupendezwa vile<br>5=Hawakupendezwa hata kidogo<br>28=DK 29=RE

102. Ni nini baadhi ya mambo unao/uliozingatia inayo/iliyo kutia mutisha ili upashwe tohara?

**102a. Kuimarika kwa usafi**	1 = Ndiyo	2 = La	28 = DK	29 = RE
**102b. Kinga kutokana na magonjwa ya zinaa/ viini vya UKIMWI**	1 = Ndiyo	2 = La	28 = DK	29 = RE
**102c. Kukubaliwa na makabila mengine**	1 = Ndiyo	2 = La	28 = DK	29 = RE
**102d. Kwa burudisho la ngono kwako**	1 = Ndiyo	2 = La	28 = DK	29 = RE
**102e. Kwa burudisho la ngono kwa washirika**	1 = Ndiyo	2 = La	28 = DK	29 = RE
**102f. Hakuna gharama**	1 = Ndiyo	2 = La	28 = DK	29 = RE
**102g. Ilipatikana kwenye kituo cha afya kilicho karibu**	1 = Ndiyo	2 = La	28 = DK	29 = RE
**102h. Ushawishi kutoka kwa marafiki/marika**	1 = Ndiyo	2 = La	28 = DK	29 = RE
**102i. Ingine, eleza**	______________________________			

103. Ni nini baadhi ya mambo gani uliyozingatia ambayo yana/yalikuuwa moyo iliusipashwe tohara?

**103a. Maumivu wakati/baada ya kupashwa tohara**	1 = Ndiyo	2 = La	28 = DK	29 = RE
**103b. Madhara yanayoweza kutokezea**	1 = Ndiyo	2 = La	28 = DK	29 = RE
**103c. Mila / Desturi / Dini**	1 = Ndiyo	2 = La	28 = DK	29 = RE
**103d. Gharama ya tohara , ikiwemo muda wa kutokuwa kazini**	1 = Ndiyo	2 = La	28 = DK	29 = RE
**103e. Kwa burudisho la ngono kwako**	1 = Ndiyo	2 = La	28 = DK	29 = RE
**103f. Kwa burudisho la ngono kwa washirika**	1 = Ndiyo	2 = La	28 = DK	29 = RE
**103g. Kituo kilikocho karibu kiko mbali**	1 = Ndiyo	2 = La	28 = DK	29 = RE
**103h. Muda mrefu wa kupona**	1 = Ndiyo	2 = La	28 = DK	29 = RE
**103i. Ingine, eleza**	______________________________			

**104. Je, utampasha mototo(wa) wako tohara?**	1 = Ndiyo	2 = La	28 = DK	29 = RE
**104a. Kama ndiyo, akiwa na umri gani?**	______________________________			

"Asante sana kwa ushirika wako. Habari uliyotupa ni ya kusaidia sana na tunakushukuru kwa wakati wako na usaidizi wako. Je, una swali la mwisho au maoni ungependa kunipa?"

"Sasa nitakuuliza habari kwa kikamilifu kuhusu kila uhusiano wa ngono uliokuwa nao kwa miezi 6 zilizopita." Elekea kwa fomu 03 Historia ya Ngono.
