# Supplementary material for: Penile coital injuries in men decline after circumcision: Results from a prospective study of recently circumcised and uncircumcised men in western Kenya
Source: PLoS One. 2017 Oct 10;12(10):e0185917. doi: 10.1371/journal.pone.0185917 (PMC5634596; doi:10.1371/journal.pone.0185917)
Supplement: S1 File — (ZIP) [file pone.0185917.s001.zip › SHABS FORM 02 - Dholuo v2.pdf]

**Namba mar kar Nonro #**	**Namba mar Nonro #**	**Limbe #**	**Tarik mar Limbe**	**Namba mar Japenjo**
_ _ _ _	_ _ _ _ _ _	_ _	_ _ / _ _ / _ _ _ _<br>dd mm y y y y	_ _

Instructions: complete the Behavioral Questionnaire Form 02 at enrollment, 6 month, 12 month, 18 months and 24 months of follow-up visits. DK = don't know, RE = refused to answer.

"Yie ipar ni ok ni nyaka ichiw duoko ne penjo ma ok idwar chiwo duoko mare kendo inyalo weyo dhi nyime gi penjo saa moro a mora."

Kidieny 1: Weche ma otudore gi Jachiwore

1. Gi e sani be in gi yo miyutogo? **1 = Ee** **2 = Ooyo** **28 = DK** **29 = RE**

Ka ee, tich mane ma itimo mamiyi yuto

1= Ondika ndalo duto

2 = Ondika kinde moko, bang' kinde matin, kata e odiechieng' ka odiechieng'

3 = Andikora kenda

Ka Ooyo:

4 = Aonge tich kendo amanyo

5 = An dichuo ma onge tich oko

6 = An japuonjre

7 = Ase dhi yueyo kata rang'ol

8 = Mamoko (ler ane): ______________________

2. Yutoni nyalo romo pesa adi ka iriwo e dwe achiel kuom kinde moromo dweche 12 mokadho? _________

3. Be ot ma idakie nitie sitima? 1 = Ee 2 = Ooyo

4. Ji adi ma idakgo? __________

5. Ji adi maluokore e kar luok ma ijatiyogo? __________

6. Pi ma ijatiyogo chiegni gi odi maromo nade?

1 = Nitie e ot (freji)
2 = Nitie oko mar ot (aluora)
3 = En wuoth matin ne dakika 10 ka iwuok e ot
4 = En wuoth mokadho dakika 10 ka iwuok e ot

7. Ekindeni idak gi ng'a?

1 = Kenda

2 = Jaot / Jaherana ma nyako

3 = Joodwa

4 = Osiepna (s)

5 = Mamoko ______________________

28 = DK

29 = RE

8. Gi e sani be isekendo?

1 = Pok akendo, aonge jahera ma adakgo → (Go to 15)

2 = Pok akendo, an gi jahera madakgo

3 = Asekendo, adak gi jaoda

4 = Asekendo, ok adak gi jaoda

28 = DK

29 = RE

9. In gi mon adi / johera ma idakgo? Mon / Johera _________

10. Otieno manyoro, be in kod jaodi kata jaherani ma idakgo ne unindo e ot achiel? 1 = Ee 2 = Ooyo 28 = DK 29 = RE

11. Kuom dweche 6 mokadho, isedhi wuoth nyadidi ma inindo oko mokadho otieno achiel? __ __ __

Kidieny 2: Mulo Remo

12. Be isegolo remo ma idhimed ng'ato? 1 = Ee 2 = Ooyo 28 = DK 29 = RE

12a. Ka ee, nyadidi kuom dweche 6 mokadho? ______________________________

13. Be osemedi remo e osiptal? 1 = Ee 2 = Ooyo 28 = DK 29 = RE

13a. Ka ee, nyadidi kuom dweche 6 mokadho? ______________________________

14. Be nyaka nene iseriwe rembi gi mar ng'ato moro? 1 = Ee 2 = Ooyo 28 = DK 29 = RE

14a. Ka ee, nyadidi kuom dweche 6 mokadho? ______________________________

14b. Yie iler matin gima ne otimore (masira, lweny, mamoko) ______________________________

15. Be osendik pien dendi (tattooed)? 1 = Ee 2 = Ooyo 28 = DK 29 = RE

15a. Ka ee, nyadidi kuom dweche 6 mokadho? ______________________________

16. Be osesari nikech wach tuo kata wach moro a mora? 1 = Ee 2 = Ooyo 28 = DK 29 = RE

16a. Ka ee, nyadidi kuom dweche 6 mokadho? ______________________________

17. Be osechuoyi sindan nikech wach moro a mora? 1 = Ee 2 = Ooyo 28 = DK 29 = RE

17a. Ka ee, nyadidi kuom dweche 6 mokadho? ______________________________

17b. Ka ee, nyadidi ma ne othiedhi ne tuoche mag nyach kuom dweche 6 mokadho? ______________________________

18. Kuom dweche 6 mokadho, be gimoro osegwari kaka sindan kata ng'adi gi pala nikech wach moro a mora? — 1 = Ee 2 = Ooyo 28 = DK 29 = RE

18a. Ka ee, nyadidi? ________________________________

18b. Ka ee, nikech weche mage? ________________________________

Section 3: Timbe mag bedo e ringruok achiel

Koro abiro penji penjo maling'ling'. Gin ma otenore kod weche teruokni kod timbeni. Par, ka wiyi kuot kata ok idwar duoko penjo moro amora, to inyalo tamori. To akwayi, ka iduoko penjo, tem bedo jadiera.

19. Be isebedo e ringruok achiel gi nyako / dhako nyaka nene? — 1 = Ee 2 = Ooyo 29 = RE (if no, go to 34)

20. Ne in gi higini adi ka ne ibedo e ringruok achiel gi dhako / nyako mokuongo? — __ __ Higini

21. Gin nyiri / mon adi ma opogore opogore ka oriwore gi chiegi / mondegi ma isebedogo e ringruok achiel e….

21a. Kinde duto mag ngimani? — __ __ __

21b. Dweche 12 mokadho? — __ __ __

21c. Dweche 6 mokadho? — __ __ __

21d. Ndalo 30 mokadho? — __ __ __

22. Be isebedo e ringruok achiel kuom dweche 6 mokadho? — 1 = Ee 2 = Ooyo 29 = RE

23. En kinde maromo nade nyaka ne ibedi e ringruok achiel mogik?

__ __ Odiechieng'

__ __ Jumbe

__ __ Dweche

__ __ Higini

24. Nyadidi ma isebedo e ringruok achiel ka oriwore gi jaodi kaachiel gi joherani mamoko:

24a. Ndalo 7 mokadho — __ __

24b. Ndalo 30 mokadho — __ __

25. Kuom dweche 6 mokadho, bende ise:

25a. Bedo e ringruok achiel ka ichiwo pesa kata mich moro? — 1 = Ee 2 = Ooyo 29 = RE

25b. Bedo e ringruok achiel gi ng'ama dhako e kinde ma en e dwe (malo)? — 1 = Ee 2 = Ooyo 29 = RE

25c. Bedo e ringruok achiel gi johera 2 kata

**mang'eny kuom ndalo 30?**	1 = Ee 2 = Ooyo 29 = RE
**26. Be isebedo e ringruok achiel gi johera 2 kata mang'eny kuom ndalo 30?**	1 = Ee 2 = Ooyo 29 = RE
**27. Mane ibedo e ringruok achiel ma ogik...**	
**27a. Be ne oyudo ka imadho kong'o?**	1 = Ee 2 = Ooyo 29 = RE
**27b. Be ne itiyo gi rabo yunga?**	1 = Ee 2 = Ooyo 29 = RE
**27c. Be ne en gi…**	1 = Jaodi 2 = Jaherani mapile<br>3 = Jaherani manyien 4 = Ochot<br>28 = DK 29 = RE
**28. Isebedo ka iketo lewi kata nang'o duong' jaodi / jaherani e kinde maromo nade?**	1 = Podi 2 = Kadichiel 3 = Seche moko<br>4 = Kinde ka kinde 5 = Saduto 28 = DK 29 = RE
**29. Isebedo ka isoyo duong'ni e sianda jaodi / jaherani e kinde ma room nade?**	1 = Podi 2 = Kadichiel 3 = Seche moko<br>4 = Kinde ka kinde 5 = Saduto 28 = DK 29 = RE
**30. Ekinde maromo nade ma iseriwori e ringruok achiel gi dhako / nyako e odiechieng' ma irome kode no?**	1 = Podi 2 = Kadichiel 3 = Seche moko<br>4 = Kinde ka kinde 5 = Saduto 28 = DK 29 = RE
**31. Ekinde maromo nade ma isebedo e ringruok achiel bang' wiro gimoro amora (machalo yath, modhi, modhi mayomo gi mamoko) e duong'ni?**	1 = Podi 2 = Kadichiel 3 = Seche moko<br>4 = Kinde ka kinde 5 = Saduto 28 = DK 29 = RE
**31a. Kapo ni iketoga, gin ang'o gini ma ijaketo?**	______________________________
**32. Ekinde maromo nade ma isebedo e ringruok achiel gi jaodi / jaherani bang' keto gimoro ei duong'ni?**	1 = Podi 2 = Kadichiel 3 = Seche moko<br>4 = Kinde ka kinde 5 = Saduto 28 = DK 29 = RE
**32a. Kapo ni iketoga, gin ang'o gini?**	______________________________
**33. Be ihero bedo e ringruok achiel gi jaherani ka duong'ne..?** (*read options*)	1 = Otwo e kinde ma ibet e ringruok achiel kode<br>2 = Otimo pi-pi ekinde ma ibet e ringruok achiel kode<br>3 = Ongo mayiero
**Ka otwo, e lony mari, en ang'o ma dhako timo mondo omi obed motwo?**	
**33a. Oyweyo duong'ne gi nanga**	1 = Ee 2 = Ooyo 28 = DK 29 = RE
**33b. Otiyo gi yiedhe mag nyaluo kod powder**	1 = Ee 2 = Ooyo 28 = DK 29 = RE
**33c. Otiyo gi gigo molosi mag duka mayudore (kaka antiseptic, sabun, gi mamoko)**	1 = Ee 2 = Ooyo 28 = DK 29 = RE
**34. Be isebedo e ringruok achiel gi wuoyi kata dichuo?**	1 = Ee 2 = Ooyo 29 = RE Ooyo →(Go to 39)

**35. Ka ee, gin yawuoyi / chuo adi ma opogore opogore?**	__ __
**36. Be isesoyo duong'ni e sianda wuoyi moro a mora?**	1 = Ee 2 = Ooyo 29 = RE
**37. Be ng'ato osesoyo duong'ne e siandani?**	1 = Ee 2 = Ooyo 29 = RE
**38. Be dichuo moro a mora oseolo pi nyodo e dhogi?**	1 = Ee 2 = Ooyo 29 = RE

Kidieny 4: Kong'o kod Tiyo gi yedhe

**39. Be isemadho gigo motimo kong'o kaka beer, spirits, chang'aa, busaa, gi mamoko kuom jumbe ang'wen mokadho?**	1 = Ee 2 = Ooyo 28 = DK 29 = RE<br>Ooyo → (Go to 42)
**40. Kinde maromo nade ma imadho gigo ma otimo kong'o kuom jumbe ang'wen mokadho? Be inyalo wacho ni…?**	1 = Madirom dichiel e odiechieng'<br>2 = Madirom dichiel e juma<br>3 = Matin ne dichiel e juma
**41. Kuom dweche 6 mokadho, kinde maromo nade ma isebedo e ringruok achiel bang' madho kong'o kata ka ne oyudo ka imetho?**	1 = Podi 2 = Seche moko 3 = Kinde ka kinde<br>4 = Saduto 28 = DK 29 = RE
**42. Ji moko osetemo tiyo gi yedhe moko ma opogore opogore. Mage kuom magi, ka nitie, ma isetemo?**	
**42a. Bhang / njaga**	1 = Ee 2 = Ooyo
**42b. Mandrax**	1 = Ee 2 = Ooyo
**42c. Valium**	1 = Ee 2 = Ooyo
**42d. Glue**	1 = Ee 2 = Ooyo
**42e. Miraa**	1 = Ee 2 = Ooyo
**42f. Kuber**	1 = Ee 2 = Ooyo
**42g. Mamoko (Ler ane)**	______________________________
**43. Ji moko osetemo chuore yath ka gitiyo gi syringe. Be isechuori yath (ka onge wach tuo)?**	1 = Ee 2 = Ooyo 28 = DK 29 = RE
**44. Kuom dweche 6 mokadho, kinde maromo nade ma isebedo e ringruok achiel ka itiyo kata bang' tiyo gi yien-gi?**	1 = Podi 2 = Seche moko 3 = Kinde ka kinde<br>4 = Saduto 28 = DK 29 = RE

Kidieny 5: Tiyo gi Rabo yunga

**45. Be isetiyo gi rabo yunga nyaka nene?**	1 = Ee 2 = Ooyo 28 = DK 29 = RE<br>Ooyo →(Go to 49)
**46. Be isebedo gi chandruok ka itiyo gi rabo yunga?**	1 = Ee 2 = Ooyo 28 = DK 29 = RE

Ka ee, en chandruok mane:

46a. Ne ok ing'eyo tiyo gi rabo yunga 1 = Ee 2 = Ooyo

46b. Rabo yunga ne oyiech 1 = Ee 2 = Ooyo

46c. Rabo yunga ne owuok ka ne iriwori e ringruok achiel 1 = Ee 2 = Ooyo

46d. Rabo yunga ne duong' ahinya 1 = Ee 2 = Ooyo

46e. Rabo yunga ne tin ahinya 1 = Ee 2 = Ooyo

46f. Mamoko (ler ane) ________________________

47. Kuom dweche 6 mokadho, be iparo ni osebedo mayot kata matek ne in mondo iyud rabo yunga?

1 = Yot ahinya 2 = Yot moromo
3 = Tek moromo 4 = Tek ahinya
28 = DK 29 = RE

48. Be esechegi in kod rabo yunga 1 = Ee 2 = Ooyo 29 = RE

Kidieny 6: Sigand Ngima mar Nyuol

"Koro daher mar penji penjo moko kuom yoregi mag nyuol. Chuo moko winjoga rem e seche magi layo, chwero tutu e duong'gi, kata nigi adhonde e aluora mar duong'gi."

50. Kuom dweche 6 mokadho, be isewinjo:

50a. Rem ka ilayo 1 = Ee 2 = Ooyo

50b. Ilayo saa ka saa 1 = Ee 2 = Ooyo

50c. Adhonde e aliora mar duong'ni 1 = Ee 2 = Ooyo

50d. Chwero tutu ka oa e duong'ni 1 = Ee 2 = Ooyo

50e. Chandruok ka ilayo (dwaro ni irit aming'a kapok lach obiro) 1 = Ee 2 = Ooyo

50f. Rem ka ibedo e ringruok achiel 1 = Ee 2 = Ooyo

50g. Chwer remo ka ibedo e ringruok achiel 1 = Ee 2 = Ooyo

50h. Rem e piny ich 1 = Ee 2 = Ooyo

50i. Puch mar dho duong'ni 1 = Ee 2 = Ooyo

51. Ka ee kuom moro a mora e 50: Be ne ebedo e ringruok achiel ka iwinjo chandruok mag ranyisigi? 1 = Ee 2 = Ooyo 28 = DK 29 = RE

52. Be ajwoga kata jathieth moro a mora osenyisiga ni in gi tuo mar nyach? 1 = Ee 2 = Ooyo 28 = DK 29 = RE

52a. Ka ee, be inyalo paro ni en tuo mar nyach mane? ________________________________

53. Be isebedo gi tuo mar nyach kuom dweche 6 mokadho? 1 = Ee 2 = Ooyo 28 = DK 29 = RE

54. Be iseyudo thieth ne tuo mar nyach nyakanene? 1 = Ee 2 = Ooyo 28 = DK 29 = RE

54a. Ka ee, nyadidi? _____________

**54b. Be ne ochuoyi sindan ka ithiedhi?**	1 = Ee 2 = Ooyo
**54c. Be ne othiedhi ne nyach kuom thuolo mar dweche 6 mokadho?**	1 = Ee 2 = Ooyo

Kidieny 7: Bedo e ringruok achiel kod mor ma okelo

**55. Kuom dweche 6 mokadho, be nitie kinde madirom jumbe 2 kata mokadho ka ne…….**	
**55a. Ionge gi siso mar bedo e ringruok achiel?**	1 = Ee 2 = Ooyo 28 = DK 29 = RE
**55b. Ok inyal winjo ndhandhu e okang' mamalo?**	1 = Ee 2 = Ooyo 28 = DK 29 = RE
**55c. Ichopo e amidimidi (climax) piyo ahinya?**	1 = Ee 2 = Ooyo 28 = DK 29 = RE
**55d. Iwinjo rem e seche ma ibedo e ringruok achiel?**	1 = Ee 2 = Ooyo 28 = DK 29 = RE
**55e. Ok ijawinjoga ndhandhu e seche ma ibedo e ringruok achiel (kata ne ok iwinj lit moro)?**	1 = Ee 2 = Ooyo 28 = DK 29 = RE
**55f. Ijabedo gi chandruok kuom chung' mar duong'ni motegno kata dhi nyime gi chung' aming'a?**	1 = Ee 2 = Ooyo 28 = DK 29 = RE
**56. Kuom dweche 6 mokadho, kinde maromo nade ma ijawinjo ka duong'ni rewni ka iriwori e ringruok achiel?**	1 = Podi 2 = Kadichiel 2 = Seche moko<br>3 = Kinde ka kinde 4 = Saduto 28 = DK 29 = RE
**57. Kuom dweche 6 mokadho, kinde maromo nade ma pien duong'ni ja gwarore, ng'adore kata ridhore ka iriwori e ringruok achiel?**	1 = Podi 2 = Kadichiel 2 = Seche moko<br>3 = Kinde ka kinde 4 = Saduto 28 = DK 29 = RE
**58. Kuom dweche 6 mokadho, kinde maromo nade ma pien duong'ni ja chwer remo ka iriwori e ringruok achiel?**	1 = Podi 2 = Kadichiel 2 = Seche moko<br>3 = Kinde ka kinde 4 = Saduto 28 = DK 29 = RE
**59. Kuom dweche 6 mokadho, e oreya mane ma inyalo keto bedoni e ringruok achiel?**	1 = Ok amor ahinya 2 = Ok amor<br>3 = Amor 4 = Amor ahinya<br>28 = DK 29 = RE
**Ka idimbo, ere kaka imor gi…**	
**59a. E okang' ma iwinjo dwaro mar bet e ringruok achiel**	1 = Ok amor ahinya 2 = Ok amor<br>3 = Amor 4 = Amor ahinya<br>28 = DK 29 = RE
**59b. Chung' (chiek) mar duong'ni**	1 = Ok amor ahinya 2 = Ok amor<br>3 = Amor 4 = Amor ahinya<br>28 = DK 29 = RE
**59c. Chung' mar duong'ni gaming'a**	1 = Ok amor ahinya 2 = Ok amor

	3 = Amor	4 = Amor ahinya
	28 = DK	29 = RE
**59d. Yot mar soyo duong'ni e duong' jaherani**	1 = Ok amor ahinya	2 = Ok amor
	3 = Amor	4 = Amor ahinya
	28 = DK	29 = RE
**59e. Kinde ka kinde mar chung' duong'ni**	1 = Ok amor ahinya	2 = Ok amor
	3 = Amor	4 = Amor ahinya
	28 = DK	29 = RE
**59f. Yot e olo pi nyodo**	1 = Ok amor ahinya	2 = Ok amor
	3 = Amor	4 = Amor ahinya
	28 = DK	29 = RE
**59g. Okang' mar rem ka ibedo e ringruok achiel**	1 = Ok amor ahinya	2 = Ok amor
	3 = Amor	4 = Amor ahinya
	28 = DK	29 = RE
**59h. Pach jaodi / jaherani e yor bedoni e ringruok achiel**	1 = Ok amor ahinya	2 = Ok amor
	3 = Amor	4 = Amor ahinya
	28 = DK	29 = RE

Kidieny 8: Paro mag Rach kod Timbe bedo e ringruok achiel

**60. Kuom dweche 6 mokadho, be iyie ni yoregi mag bedo e ringruok achiel ose:**	1 = Dok chien	→Go to 61
	2 = Medore	→Go to 62
	3 = Odong' machal	→Go to 63
	28 = DK	→Go to 63
	29 = RE	→Go to 63

61. Gin weche mage ma omiyo yoregi mag bedo e ringruok achiel osedok chien kuom dweche 6 mokadho?

**61a. Luoro mar kute mag ayaki / tuoche mag nyae**	1 = Ee 2 = Ooyo
**61b. Bedo gi thuolo matin mar riwruok e ringruok achiel**	1 = Ee 2 = Ooyo
**61c. Aole ma owuok kuonde fwambo weche, jogweng', osiepe, gi mamoko**	1 = Ee 2 = Ooyo
**61d. Yiero ma mara mondo aduok chien yore mag bedo e ringruok achiel**	1 = Ee 2 = Ooyo
**61e. Mamoko (ler ane)**	______________________

62. **Gin weche mage ma omiyo yoregi mag bedo e ringruok achiel osemedore kuom dweche 6 mokadho?**

**62a. Medruok kuom gombo mar bedo e ringruok achiel**	1 = Ee 2 = Ooyo
**62b. Bedo gi thuolo mang'eny mar bedo e ringruok acheil bedo e ringruok ling)**	1 = Ee 2 = Ooyo
**62c. Aole ma owuok kuonde fwambo weche, jogweng', osiepe, gi mamoko**	1 = Ee 2 = Ooyo
**62d. Tero nyange**	1 = Ee 2 = Ooyo
**62e. Mamoko (ler ane)**	________________________________

63. En ang'o ma omiyo iparo ni in gi thuolo mar gamo kute mag ayaki?

1 = Onge thuol kata matin
2 = Nitie thuolo matin
3 = Nitie thuolo moromo →Go to 65
4 = Nitie thuolo mang'eny →Go to 65
5 = Akiya →Go to 66

64. Ang'o ma omiyo iparo ni ionge thuolo chutho kata thuolo matin mar gamo kute mag ayaki?

When answered, go to 66

**64a. Ok abed e ringruok achiel**	1 = Ee 2 = Ooyo
**64b. Kinde duto ti gi rabo yunga**	1 = Ee 2 = Ooyo
**64c. Bed kod jahera achiel**	1 = Ee 2 = Ooyo
**64d. Ne ni ok imedo kar kwan mag joherani**	1 = Ee 2 = Ooyo
**64e. Jaherani ogeno man an**	1 = Ee 2 = Ooyo
**64f. Tero nyange**	1 = Ee 2 = Ooyo
**64g. Mamoko (ler ane)**	________________________________

65. Ang'o ma omiyo iparo ni in gi thuolo moromo kata mang'eny mar gamo kute mag ayaki?

**65a. Ok ati gi rabo yunga**	1 = Ee 2 = Ooyo
**65b. Bedo kod jahera moloyo achiel**	1 = Ee 2 = Ooyo
**65c. Jaherana nigi johera moko**	1 = Ee 2 = Ooyo
**65d. Homosexual contacts**	1 = Ee 2 = Ooyo
**65e. Meda remo / chuowo sindan**	1 = Ee 2 = Ooyo
**65f. Ne otera nyange**	1 = Ee 2 = Ooyo
**65g. Mamoko (ler ane)**	____________________________

66. En tim mane, kaluwore gi pachi, manyalo keto ng'ato e thuolo mamalo mar gamo kute mag ayaki?

**66a. Bedo e ringruok achiel e yor duong' dhako kata bedo e ringruok achiel e yor sianda?**	1 = Bedo e ringruok achiel e yor duong' dhako maonge rageng' en thuolo mamalo<br>2 = Bedo e ringruok achiel e yor sianda maonge rageng' en thuolo mamalo<br>3 = Duto nigi thuolo mamalo machal kuom gamo kute mag ayaki<br>28 = DK 29 = RE
**66a. Bedo e ringruok achiel e yo motwo (ka jaherani oyueyo duong'ne gi nanga kata gin moro) kata motimo pi-pi?**	1 = Bedo e ringruok achiel e yo motimo pi-pi maonge regeng' en thuolo mamalo<br>2 = Bedo e ringruok achiel e yo motwo maonge regeng' en thuolo mamalo<br>3 = Duto nigi thuolo mamalo machal kuom gamo kute mag ayaki<br>28 = DK 29 = RE

67. Be iyie gi andike moluwogi?

**67a. Kawo kinda mang'eny mondo iritri eyo makare ne timbe mag bedo e ringruok achiel.**	1 = Ayie	2 = Aonge gi ng'eyo	3 = Ok ayie
**67b. Iwinjo oolo kinde duto e ng'iyo timbegi mag bedo e ringruok achiel.**	1 = Ayie	2 = Aonge gi ng'eyo	3 = Ok ayie
**67c. Ka imer, yot ahinya mondo ibed e ringruok achiel gi ji moko ma opogore gi jaherani mapile.**	1 = Ayie	2 = Aonge gi ng'eyo	3 = Ok ayie
**67d. Ka imer, yot ahinya mondo ibed e ringruok achiel maonge rabo yunga.**	1 = Ayie	2 = Aonge gi ng'eyo	3 = Ok ayie

Kidieny 9: Paro kuom bedo e ringruok achiel kod terruok

68. Yie inyisa ka iyie kata ok iyie gi andike ma oluwogo.

**68a. Chuo nyalo winjo mor eyor bedo e ringruok achiel kata mana gi johera ma ok gihero**	1 = Ayie ahinya 2 = Ayie 3 = Aonge gi ng'eyo 4 = Ok ayie 5 = Ok ayie kata matin 29 = RE
**68b. Dichuo ma "dichuo" oyikore seche duto eyor bedo e ringruok achiel**	1 = Ayie ahinya 2 = Ayie 3 = Aonge gi ng'eyo 4 = Ok ayie 5 = Ok ayie kata matin 29 = RE
**68c. Mon nigi thuolo mamalo mar tamo gombo mag bedo e ringruok achiel moloyo jomachuo**	1 = Ayie ahinya 2 = Ayie 3 = Aonge gi ng'eyo 4 = Ok ayie 5 = Ok ayie kata matin 29 = RE
**68d. Bedo e ringruok achiel en gima duong' ahinya e ngima**	1 = Ayie ahinya 2 = Ayie 3 = Aonge gi ng'eyo 4 = Ok ayie 5 = Ok ayie kata matin 29 = RE
**68e. Koso bedo gi nyalo mar bet e ringruok achiel en kuyo ne johera**	1 = Ayie ahinya 2 = Ayie 3 = Aonge gi ng'eyo 4 = Ok ayie 5 = Ok ayie kata matin 29 = RE
**68f. Rang'iny mar chung' motegno mar duong' dichuo ema romo mon**	1 = Ayie ahinya 2 = Ayie 3 = Aonge gi ng'eyo 4 = Ok ayie 5 = Ok ayie kata matin 29 = RE
**68g. Dichuo ma "dichuo' bet e ringruok achiel kinde ka kinde**	1 = Ayie ahinya 2 = Ayie 3 = Aonge gi ng'eyo 4 = Ok ayie 5 = Ok ayie kata matin 29 = RE
**68h. Mon ma bergi ok ywa wang' e yor bedo e ringruok achiel ok nyal winjo ka giromo ka obed kodgi e ringruok achiel**	1 = Ayie ahinya 2 = Ayie 3 = Aonge gi ng'eyo 4 = Ok ayie 5 = Ok ayie kata matin 29 = RE
**68i. Amor kod chuech mar denda kaka chalo**	1 = Ayie ahinya 2 = Ayie 3 = Aonge gi ng'eyo 4 = Ok ayie 5 = Ok ayie kata matin 29 = RE

Kidieny 10: Paro kuom Nyange

**69. Yot mondo irit duong'ni obed maler ka…..?**	1 = Otera nyange 2 = Ok otera nyange 3 = Onge pogruok 28 = DK 29 = RE
**70. Yot mondo iyud tuo ka owuok kuom dhako ka…?**	1 = Otera nyange 2 = Ok otera nyange 3 = Onge pogruok 28 = DK 29 = RE
**71. Yot mondo iyud kute mag ayaki ka…?**	1 = Otera nyange 2 = Ok otera nyange 3 = Onge pogruok 28 = DK 29 = RE

72. Chuo winjo ndhandhu moloyo e seche ma giriwore e ringruok achiel ka…?

1 = Otergi nyange 2 = Ok otergi nyange
3 = Onge pogruok 28 = DK 29 = RE

73. Thoth mon winjo ndhandhu moloyo ka giriwore e ringruok achiel gi chuo ma…?

1 = Oter nyange 2 = Ok oter nyange
3 = Onge pogruok 28 = DK 29 = RE

74. Chuo ma ….. ohero bedo e ringruok achiel

1 = Oter nyange 2 = Ok oter nyange
3 = Onge pogruok 28 = DK 29 = RE

75. Ikwayi ni imiwa pachi kuom tero nyange:

**75a. Koro kaka tero nyange yudore, kute mag ayaki koro ok en gima lich ahinya moloyo kaka ne en tie chon.**	1 = Ayie	2 = Aonge gi ng'eyo	3 = Ok ayie
**75b. Koro kaka tero nyange yudore, tiyo gi rabo yunga ok en gima koro ochuno.**	1 = Ayie	2 = Aonge gi ng'eyo	3 = Ok ayie
**75c. Koro kaka tero nyange yudore, ok aparra ahinya ne weche kute mag ayaki.**	1 = Ayie	2 = Aonge gi ng'eyo	3 = Ok ayie
**75d. Koro kaka tero nyange yudore, yot ahinya mondo koro abed kod johera mang'eny moloyo achiel.**	1 = Ayie	2 = Aonge gi ng'eyo	3 = Ok ayie
**75e. Koro kaka tero nyange yudore, an gi ikruok mar chiwora kuom gamo kute mag ayaki kata chiwo kutegi ne ng'at machielo.**	1 = Ayie	2 = Aonge gi ng'eyo	3 = Ok ayie
**75f. Koro kaka tero nyange yudore, ng'ato mantie gi kute mag ayaki ok onego parore ahinya kuom tiyo gi rabo yunga.**	1 = Ayie	2 = Aonge gi ng'eyo	3 = Ok ayie
**75g. Koro kaka tero nyange yudore, in gi thuolo mang'eny mar bedo e ringruok achiel maonge rabo yunga.**	1 = Ayie	2 = Aonge gi ng'eyo	3 = Ok ayie

Kidieny 11: Ler

76. Kinde ka kinde maromo nade ma ijaluoko duong'ni?

1 = Dichiel e dwe kata matin 2 = Ding'eny e dwe
3 = Juma ka juma 4 = Ding'eny e juma
5 = Pile 6 = Ok oluok duong'na

77. Mane ibedo e ringruok achiel gi nyako/dhako mogik be ne iluoko duong'ni e sechego bang' tieko?

1 = Ee 2 = Ooyo 28 = DK 29 = RE

Ang'o mane itiyogo kuom keto duong'ni obed maler?

**77a. Nanga/otamba maonge pi**	1 = Ee	2 = Ooyo	28 = DK	29 = RE
**77b. Pi**	1 = Ee	2 = Ooyo	28 = DK	29 = RE
**77c. Sabun gi pi**	1 = Ee	2 = Ooyo	28 = DK	29 = RE
**77d. Yiedhi nyaluo**	1 = Ee	2 = Ooyo	28 = DK	29 = RE

77e. Mamoko (ler ane) ______________________________

77f. Ne okawi kinde maromo nade mondo iliok duong'ni bang' bedo e ringruok achiel? _______Saa _____ Dakika

78. Be iseketo kata wiro gimoro e wi duong'ni nikech wach moro a mora? 1 = Ee 2 = Ooyo 28 = DK 29 = RE →Go to next section

78a. Ka ee, ang'o ma omiyo iwiro gigo?

1 = Ne ler / nego kute
2 = Gelo muya marach
3 = Komo nyuol
4 = Thidho ilo
5 = Bang' bedo achiel e ringruok mondo duong' obed maler
6 = Kapok bedo e ringruok achiel ochakore/ikruok
7 = Thiedho adhonde
8 = Mondo ageng'ora ne tuo/yamo makelo tuo
9 = mondo ageng' jaherana kuom gamo tuo/yamo makelo tuo
29 = RE

Kidieny 12: Pimo kute mag Ayaki kod Hocho

79. Be osetimni pim mar kute mag ayaki nyaka nene? 1 = Ee 2 = Ooyo 28 = DK 29 = RE

80. Ka ne otimni pim mar kute mag ayaki, be ne iyudo weche korka duoko thuolo mar gamo kute mag ayaki? 1 = Ee 2 = Ooyo 28 = DK 29 = RE

81. Osetimni pim mar kute mag ayaki didi? __ __

82. Ne en karang'o mane otimni pim mogik? 1 = Matin ne higa achiel 2 = Ekind higa 1-2 mokalo 3 = Okadho higini 2

83. Adhi mondo apenji kuom duoko mar pimni mar kute mag ayaki, to inyalo tamori duoko ka ihero. Duoko mogik mar pimni ne kute mag ayaki en mane?

1 = Positive 2 = Negative (go to 86)
3 = Ok ne ayudo duoko (go to 86)
29 = RE (go to 86)

84. Be idhiga e kar thieth mondo iyud thieth ne kute mag ayaki? 1 = Ee 2 = Ooyo 28 = DK 29 = RE

85. Be iyudo yiedhe magayo landruok ne kute mag ayaki (ART)? 1 = Ee 2 = Ooyo 28 = DK 29 = RE

Kidieny 13: Nyange

**86. Be oseteri nyange?**	1 = Ee 2 = Ooyo → Go to 103
**87. Ne oteri nyange kanye?**	1 = E kar thieth kae<br>2 = Mamoko (ler ane)______________________
**88. Ne oteri nyange chon maromo nade?**	__ __ Odiechieng'<br>__ __ Jumbe<br>__ __ Dweche
**89. Imor maromo nade ….**	
**89a. Gi yo mane oterie nyange?**	1 = Amor ahinya 2 = Amor moromo<br>3 = Onge gima dawachi 4 = Ok amor<br>5 = Ok amor kata matin 28 = DK 29 = RE
**89b. Gi kaka duong'ni nenore (chalo)?**	1 = Amor ahinya 2 = Amor moromo<br>3 = Onge gima dawachi 4 = Ok amor<br>5 = Ok amor kata matin 28 = DK 29 = RE
**89c. Gi nyalo mar bedo e ringruok achiel?**	1 = Amor ahinya 2 = Amor moromo<br>3 = Onge gima dawachi 4 = Ok amor<br>5 = Ok amor kata matin 28 = DK 29 = RE
**90. Be isebedo gi chung' mar duong' motegno nyaka ne teri nyange?**	1 = Ee 2 = Ooyo 29 = RE
**90a. Ka ee, be chung' duong'ni en kaka pile?**	1 = Ee 2 = Ooyo 29 = RE
**90a.1 Ka ooyo, ang'o ma omiyo?**	
**Oremo**	1 = Ee 2 = Ooyo 29 = RE
**Ochwero remo**	1 = Ee 2 = Ooyo 29 = RE
**Iwinjo ka oridore/oyware**	1 = Ee 2 = Ooyo 29 = RE
**90b. Ka ee ne 90, ka ipimo gi ka ne pok oteri nyange, chung' duong'ni otegno maromo nade**	1 = Matek 2 = Matek matin 3 = Bet chalre 29 = RE
**91. Ka opogore gi kinde ma duong'ni ne chango, be isegaweyo ma ok ibet e ringruok achiel nikech oteri nyange?**	1 = Ee 2 = Ooyo 28 = DK 29 = RE
**92. Be isebet e ringeuok achiel nyaka ne yang'i?**	1 = Ee 2 = Ooyo 29 = RE →Go to 98f
**93. Mapiyo maromo nade bang' yeng'o mane ibedo e ringruok achiel?**	__ __ Seche<br>__ __ Odiechienge<br>__ __ Jumbe

	__ __ Dweche
**94. Be isetiyo gi rabo yunga bang'e ka oseteri nyange?**	1 = Ee 2 = Ooyo 29 = RE →Go to 96
**95. Be ne iyudo ka tiyo gi rabo yunga yot bang' nyange, ka ipimo gi kinde ma pok ne oteri nyange?**	1 = Yot moloyo bang' ka osetera nyange<br>2 = Yot moloyo kane pok otera nyange<br>3 = Onge pogruok<br>4 = Ok ne ati gi rabo yunga kane pok otera nyange<br>28 = DK<br>29 = RE
**96. Be iparo ni ne iwinjo mamit ahinya ka ibet e ringruok achiel kapok ne oteri nyange kose bang'e ka oseteri nyange?**	1 = Awinjo mit ahinya kane pok otera nyange<br>2 = Awinjo mit ahinya bang' ka osetera nyange<br>3 = Onge pogruok<br>28 = DK<br>29 = RE
**97. Be iparo ni ne iwinjo mamit ahinya ka ibet e ringruok achiel kapok ne oteri nyange kose bang'e ka oseteri nyange ne ja/joherani)?**	1 = En mit ahinya ne joherana bang' tera nyange<br>2 = En mit ahinya ne joherana kane pok otera nyange<br>3 = Onge pogruok<br>28 = DK<br>29 = RE

98. Ka ipimo kod ndalo mane pok oteri nyange:

**98a. Inyalo wacho ni duong'ni winjo ndhandhu maromo nade?**	1=Mang'eny 2=Mabet ng'eny<br>3=Chal a chala 4=Odok piny matin<br>5=Odok piny ahinya 28=DK 29=RE
**98b. Inyalo wacho ni ichopo e amidimidi mayot maromo nade ka ibet e ringruok achiel?**	1=Mang'eny 2=Mabet ng'eny<br>3=Chal a chala 4=Odok piny matin<br>5=Odok piny ahinya 28=DK 29=RE
**98c. Ijabet e ringruok achiel kinde ka kinde maromo nade?**	1=Mang'eny 2=Mabet ng'eny<br>3=Chal a chala 4=Odok piny matin<br>5=Odok piny ahinya 28=DK 29=RE
**98d. Be ijabedo gi garruok, yiech kata ng'adruok mang'eny, matin, kata chal a chala?**	1=Mang'eny 2=Mabet ng'eny<br>3=Chal a chala 4=Odok piny matin<br>5=Odok piny ahinya 6= Pok abedo gi garruok a chon<br>28=DK 29=RE

**98e. Be iolo pi nyodo mapiyo ma opogore gi dwaroni?**	1=Mang'eny 2=Mabet ng'eny<br>3=Chal a chala 4=Odok piny matin<br>5=Odok piny ahinya 6= Did not experience early ejaculation before<br>28=DK 29=RE
**98f. Iparo ni igeng'ori moromo nade ne tuoche mag nyae?**	1=Mang'eny 2=Mabet ng'eny<br>3=Chal a chala 4=Odok piny matin<br>5=Odok piny ahinya 28=DK 29=RE
**98g. E seche ma ilayo ineno ka ober, orach kata chal a chala?**	1=Ber ahinya 2=Ber moromo<br>3=Chal a chala 4=Rach moromo<br>5=Rach ahinya 28=DK 29=RE
**99. Be teri nyange oseloko timbegi mag bedo e ringruok achiel eyo moro a mora?**	1 = Ee 2 = Ooyo 28 = DK 29 = RE
**100. Nyaka ne teri nyange, ere kaka ber mar mon oseywayi?**	1=Mang'eny moloyo 2=Mabet ng'eny<br>3=Chal a chala 4=Odok piny matin<br>5=Odok piny ahinya 28=DK 29=RE
**101. Ka opogore gi joheragi ma ibedogo e ringruok achiel, be nitie ng'ato e odu ma ong'eyo ni oseteri nyange?**	1 = Ee 2 = Ooyo 28 = DK 29 = RE
**101a. Ka ee, eyore duto, pachgi en mane?**	1=Gimor ahinya<br>2=Gimor moromo<br>3=Gin kanyo kata onge paro ma gichiwo<br>4=Ok gimor moromo<br>5=Ok gimor ahinya<br>28=DK<br>29=RE
**102. Be jaherani moro a mora oseng'eyo ni oteri nyange?**	1 = Ee 2 = Ooyo 28 = DK 29 = RE
**102a. Ka ee, be ne gimor kata ok gimor?**	1=Gimor ahinya<br>2=Gimor moromo<br>3=Gin kanyo kata onge paro ma gichiwo<br>4=Ok gimor moromo<br>5=Ok gimor ahinya<br>28=DK 29=RE
**103. Gin weche mage mane nyalo/ne ojiwi mondo oteri nyange?**	
**103a. Ler mamalo mar del**	1 = Ee 2 = Ooyo 28 = DK 29 = RE
**103b. Geng'ruok ne tuoche mag nyae/kute mag ayaki**	1 = Ee 2 = Ooyo 28 = DK 29 = RE
**103c. Bedo ni dhoute moko moko oyie kode**	1 = Ee 2 = Ooyo 28 = DK 29 = RE

**103d. Ndhandhu mar bet e ringruok achiel ne an**	1 = Ee 2 = Ooyo 28 = DK 29 = RE
**103e. Ndhandhu mar bet e ringruok achiel ne joherana**	1 = Ee 2 = Ooyo 28 = DK 29 = RE
**103f. Onge chudo**	1 = Ee 2 = Ooyo 28 = DK 29 = RE
**103g. Ne oyudore e kar thieth mae gweng'**	1 = Ee 2 = Ooyo 28 = DK 29 = RE
**103h. Aole mag osiepe / mbese**	1 = Ee 2 = Ooyo 28 = DK 29 = RE
**103i. Mamoko, ler ane**	______________________

104. Gin weche mage ma ok nyal/tami mondo oteri nyange?

**104a. Rem e seche/bang' teri nyange**	1 = Ee 2 = Ooyo 28 = DK 29 = RE
**104b. Rach kata chwanyruok manyalo betie**	1 = Ee 2 = Ooyo 28 = DK 29 = RE
**104c. Culture / Tradition / Religion**	1 = Ee 2 = Ooyo 28 = DK 29 = RE
**104d. Chudo mar yeng'o, ka oriwore gi thuolo mar yueyo ka ok adhi tich**	1 = Ee 2 = Ooyo 28 = DK 29 = RE
**104e. Ndhandhu mar bet e ringruok achiel ne an**	1 = Ee 2 = Ooyo 28 = DK 29 = RE
**104f. Ndhandhu mar bet e ringruok achiel ne joherana**	1 = Ee 2 = Ooyo 28 = DK 29 = RE
**104g. Kar thieth machiegni nitie mabor**	1 = Ee 2 = Ooyo 28 = DK 29 = RE
**104h. Kinde malach mar chango**	1 = Ee 2 = Ooyo 28 = DK 29 = RE
**104i. Mamoko, ler ane**	______________________

**105. Be inyalo tero wuodi/yawuoti nyange?**	1 = Ee 2 = Ooyo 28 = DK 29 = RE
**105a. Ka ee, ka en jahigini adi?**	______________________

"Erokamano kuom miyowa yot kuom tiyo kodwa. Weche ma imiyowa biro konyowa ahinya kendo wapuoyo thuolono kod kony mimiyowa. Be dibedie gi penjo moro mogik kata wache ma diher pimo kodwa?"

"Abiro koro penji weche maiye kuom bedoni e ringruok achiel moro ka moro mane itimo kuom dweche 6 mokadho." Go to Form 03 Sexual History
