# Supplementary material for: Penile coital injuries in men decline after circumcision: Results from a prospective study of recently circumcised and uncircumcised men in western Kenya
Source: PLoS One. 2017 Oct 10;12(10):e0185917. doi: 10.1371/journal.pone.0185917 (PMC5634596; doi:10.1371/journal.pone.0185917)
Supplement: S1 File — (ZIP) [file pone.0185917.s001.zip › SHABS FORM 01 - Kiswahili v2.pdf]

SHABS	**HABARI KUMHUSU MSHIRIKI**<br>**Version 2 / February 06, 2008**	Fomu 01<br>**Ukurasa la 1 ya 1**

**Nambari ya Kituo #**	**Nambari ya Kushiriki #**	**Nambari ya Kuzuru#**	**Tarehe ya Kuzuru**	**Kitambulishi cha Anayehoji**
_ _ _ _	_ _ _ _ _ _	_ _	_ _ / _ _ / _ _ _ _<br>dd mm y y y y	_ _

Maagizo: Jaza habari kumuhusu mshiriki katika Fomu 01 wakati wa kusajiliwa pekee DK = sijui know, RE = amekataa kujibu. **"Tafadhali kumbuka kuwa si lazima ujibu maswali ambayo hungependa kuyajibu na waweza kukatiza mahojiano wakati wowote."**

1. Tarehe ya kuzaliwa ______/______/_ _ _ _
tarehe mwezi mwaka

2. Wakati ulisherekea siku ya kuzaliwa kwako mwisho, ulikuwa na umri gani? ___________

3. Wilaya yako ni ipi?

1 = Kisumu Mashariki	2 = Kisumu Magharibi	3 = Siaya	4 = Nyando	5 = Rachuonyo
6 = Bondo	7 = Migori	8 = Suba	9 = Kisii	10 = Gucha
11 = Nyamira	12 = Kuria	13 = Homa Bay	14 = Rarieda	15 = Vihiga
16 = Borabu	17 = Kakamega	18 = Nandi	19 = Kericho	20 = Ingine
21 = Butere/Mumias	22 = Busia	28 = DK	29 = RE	

4. Dini yako ni gani?

1 = Katoliki	2 = Kianglikana	3 = Muisilamu	4 = Pentekosti	5 = 7th Day Advantist
6 = Ingine	7 = Hauna dini	28 = DK	29 = RE	

5. Kabila lako ni gani?

1 = Mjaluo	2 = Mkikuyu	3 = Mkisii	4 = Mkalenjin	5 = Mkamba
6 = Mluhya	7 = Mmeru/Membu	8 = Mmaasai	9 = Mkuria	10 = Pwani
11 = Other				

6. Umetimiza miaka ngapi shuleni? ___________

7. Je, waweza kusoma gazeti au barua? 1 = Ndiyo 2 = Zingine 3 = La 28 = DK 29 = RE

8. Unaishi wapi kwa wakati huu?

**Taarafa za wilaya ya Kisumu:**	1 = Kombewa	2 = Maseno	3 = Winam	4 = Kadibo
**Taarafa za wilaya ya Nyando:**	5 = Lower Nyakach	6 = Miwani	7 = Muhoroni	8 = Nyando (Awasi)
	9 = Upper Nyakach			

10 = Ingine wilaya – Eleza ______________

9. Umeishi hapa kwa muda gani?

Miaka ___ ___ Miezi ___ ___

[kama ni chini ya mwezi mmoja, andika miezi 00, kama ni milele, andika miaka 98]
