# Supplementary material for: Penile coital injuries in men decline after circumcision: Results from a prospective study of recently circumcised and uncircumcised men in western Kenya
Source: PLoS One. 2017 Oct 10;12(10):e0185917. doi: 10.1371/journal.pone.0185917 (PMC5634596; doi:10.1371/journal.pone.0185917)
Supplement: S1 File — (ZIP) [file pone.0185917.s001.zip › SHABS FORM 01 - Dholuo v2.pdf]

SHABS	**WECHE JOCHIWORE**<br>**Version 2 / February 06, 2008**	Form 01<br>**Page 1 of 1**

**Namba mar kar timo nonro #**	**Namba mar nonro #**	**Limbe #**	**Tarik mar limbe**	**Namba japenj penjo**
_ _ _ _	_ _ _ _ _ _	_ _	_ _ / _ _ / _ _ _ _<br>dd mm yyyy	_ _

Siem: Pong' oboke mar 01 e limbe mar donjo e nonro kende. DK= Kia, RE= Otamore dwoko **"Kiyie to par ni ok ochuno ni nyaka idwok penjo ka ok idwar dwoko kendo inyalo weyo duoko penjo e sa asaya."**

1. Tarik mar nyuol _ _/_ _/_ _ _ _
dd mm yyyy

2. Ne in ja higni adi e tarik mar nyuolni mogik? ___________

3. District ni mar nyuol en mane?

1 = Kisumu East	2 = Kisumu West	3 = Siaya	4 = Nyando	5 = Rachuonyo
6 = Bondo	7 = Migori	8 = Suba	9 = Kisii	10 = Gucha
11 = Nyamira	12 = Kuria	13 = Homa Bay	14 = Rarieda	15 = Vihiga
16 = Borabu	17 = Kakamega	18 = Nandi	19 = Kericho	20 = Other
21 = Butere/Mumias	22 = Busia	28 = DK	29 = RE	

4. Din mari en mane?

1 = Catholic	2 = Anglican	3 = Muslim	4 = Pentecostal	5 = 7th Day Advantist
6 = Mamoko	7 = Onge din	28 = DK	29 = RE	

5. In ja dhok mane?

1 = Luo	2 = Kikuyu	3 = Kisii	4 = Kalenjin	5 = Kamba
6 = Luhya	7 = Meru/Embu	8 = Maasai	9 = Kuria	10 = Coastal
11 = Mamoko				

6. Idhi skul nyaka rang'iny mane? ___________

7. Bende inyalo somo gazet? 1 = Ee 2 = Moko 3 = Ooyo 28 = DK 29 = RE

8. Sani idak kanye?

**Divisions mag Kisumu District:**	1 = Kombewa	2 = Maseno	3 = Winam	4 = Kadibo
**Divisions mag Nyando District:**	5 = Lower Nyakach	6 = Miwani	7 = Muhoroni	8 = Nyando (Awasi)
	9 = Upper Nyakach			

10 = District mamoko – Ler tiende ______________

9. Ise dak kae kuom thuolo maromo nade?

Higni___ ___ Dweche ___ ___
[Katin ne dwe achiel ndik00. Ka nyaka nene ndik higni 98.]
