# Supplementary material for: Penile coital injuries in men decline after circumcision: Results from a prospective study of recently circumcised and uncircumcised men in western Kenya
Source: PLoS One. 2017 Oct 10;12(10):e0185917. doi: 10.1371/journal.pone.0185917 (PMC5634596; doi:10.1371/journal.pone.0185917)
Supplement: S1 File — (ZIP) [file pone.0185917.s001.zip › SHABS FORM 01 - English v2 Demographics.pdf]

SHABS	**DEMOGRAPHICS**<br>**Version 2 / February 06, 2008**	Form 01<br>**Page 1 of 1**

**Site ID #**	**Study ID #**	**Visit #**	**Visit Date**	**Interviewer ID**
_ _ _ _	_ _ _ _ _ _	_ _	_ _ / _ _ / _ _ _ _<br>dd mm yyyy	_ _

Instructions: Complete Demographic Form 01 at the enrollment visit only DK = don't know, RE = refused to answer. **"Please remember that you do not have to answer any questions that you do not want to answer and you may discontinue the interview at any time."**

1. Date of birth _ _/_ _/_ _ _ _
dd mm yyyy

2. How old were you on your last birthday? ___________

3. What is your home district?

1 = Kisumu East	2 = Kisumu West	3 = Siaya	4 = Nyando	5 = Rachuonyo
6 = Bondo	7 = Migori	8 = Suba	9 = Kisii	10 = Gucha
11 = Nyamira	12 = Kuria	13 = Homa Bay	14 = Rarieda	15 = Vihiga
16 = Borabu	17 = Kakamega	18 = Nandi	19 = Kericho	20 = Other
21 = Butere/Mumias	22 = Busia	28 = DK	29 = RE	

4. What is your religion?

1 = Catholic	2 = Anglican	3 = Muslim	4 = Pentecostal	5 = 7th Day Advantist
6 = Other	7 = No religion	28 = DK	29 = RE	

5. What is your ethnic group/tribe?

1 = Luo	2 = Kikuyu	3 = Kisii	4 = Kalenjin	5 = Kamba
6 = Luhya	7 = Meru/Embu	8 = Maasai	9 = Kuria	10 = Coastal
11 = Other				

6. How many years of school have you attained? ___________

7. Can you read a newspaper or a letter? 1 = Yes 2 = Some 3 = No 28 = DK 29 = RE

8. Where are you currently staying?

**Kisumu Districts divisions:**	1 = Kombewa	2 = Maseno	3 = Winam	4 = Kadibo
**Nyando District divisions:**	5 = Lower Nyakach	6 = Miwani	7 = Muhoroni	8 = Nyando (Awasi)
	9 = Upper Nyakach			

10 = Other district – Specify ______________

9. How long have you stayed there?

Years ___ ___ Months ___ ___
[If less than one month, enter 00 months. If forever, enter 98 years.]
