# Supplementary material for: Penile coital injuries in men decline after circumcision: Results from a prospective study of recently circumcised and uncircumcised men in western Kenya
Source: PLoS One. 2017 Oct 10;12(10):e0185917. doi: 10.1371/journal.pone.0185917 (PMC5634596; doi:10.1371/journal.pone.0185917)
Supplement: S1 File — (ZIP) [file pone.0185917.s001.zip › SHABS FORM 02 - English Behavioral Questionnaire v2.pdf]

**Site ID #**	**Study ID #**	**Visit #**	**Visit Date**	**Interviewer ID**
_ _ _ _	_ _ _ _ _ _	_ _	_ _ / _ _ / _ _ _ _<br>dd mm y y y y	_ _

Instructions: complete the Behavioral Questionnaire Form 02 at enrollment, 6 month, 12 month, 18 months and 24 months of follow-up visits. DK = don't know, RE = refused to answer.

"Please remember that you do not have to answer any questions that you do not want to answer and you may discontinue the interview at any time."

Section 1: Background Characteristics

1. Are you currently employed? **1 = Yes** **2 = No** **28 = DK** **29 = RE**

If Yes, what do you currently do to earn a living

1= Regularly employed
2 = Employed seasonally, on short-term contract, or on a day-to-day basis
3 = Self-employed

If No:

4 = Unemployed and looking for work
5 = A homemaker with no other work outside
6 = A student
7 = Retired or disabled
8 = Other (specify): ______________________

2. What is your average monthly income over the last 12 months? ___________

3. Do you have electricity in your household? 1 = Yes 2 = No

4. How many people stay with you in your household?__________

5. With how many people do you share your bathing room?__________

6. Where is the closest to your house source of water?

1 = In house (tap water)
2 = Right outside of house (in your compound)
3 = Less than 10 minutes walk from your house
4 = More than 10 minutes walk from your house

7. With whom do you currently live?

1 = Alone
2 = Wife/live-in female partner
3 = Family
4 = Friend(s)

	5 = Other ____________________
	28 = DK
	29 = RE
**8. What is your current marital status?**	1 = Single, without live-in partner →(Go to 11)
	2 = Single, with live-in partner
	3 = Married, living with wife
	4 = Married, not living with wife
	28 = DK
	29 = RE
**9. How many wives/live-in partners do you have?**	Wives / partners _________
**10. Last night, did you and a wife or live-in partner sleep in the same house?**	1 = Yes 2 = No 28 = DK 29 = RE
**11. In past 6 months, how many trips of more than one night did you have?**	__ __ __

Section 2: Blood Exposure

**12. Have you ever donated blood?**	1 = Yes 2 = No 28 = DK 29 = RE
**12a. If yes, how many times in last 6 months?**	______________________________
**13. Have you ever received blood in a hospital (a blood transfusion)?**	1 = Yes 2 = No 28 = DK 29 = RE
**13a. If yes, how many times in last 6 months?**	______________________________
**14. Have you ever come in contact with blood of another person?**	1 = Yes 2 = No 28 = DK 29 = RE
**14a. If yes, how many times in last 6 months?**	______________________________
**14b. Please briefly state what happened (accident, fight, etc.)**	______________________________
**15. Have you ever been tattooed?**	1 = Yes 2 = No 28 = DK 29 = RE
**15a. If yes, how many times in last 6 months?**	______________________________
**16. Have you ever practiced bloodletting (saro) for health or other purposes?**	1 = Yes 2 = No 28 = DK 29 = RE
**16a. If yes, how many times in last 6 months?**	______________________________
**17. Have you ever received an injection for any reason?**	1 = Yes 2 = No 28 = DK 29 = RE

17a. If yes, how many times in last 6 months? ______________________________

17b. If yes, how many of them were for STD treatment in last 6 months? ______________________________

18. In the last 6 months, have you been pricked by a needle or cut by a knife for any reason? 1 = Yes 2 = No 28 = DK 29 = RE

18a. If yes, how many times? ______________________________

18b. If yes, what were some of the reasons? ______________________________

Section 3: Sexual practices

Now I am going to ask you questions that are a bit more sensitive. They are about your sexual relations and practices. Remember, if you are embarrassed or do not want to answer any question, you may refuse to answer. But, if you do give an answer please be truthful.

19. Have you ever had vaginal sex with a girl/woman? 1 = Yes 2 = No 29 = RE (if no, go to 34)

20. How old were you (in years) when you had sex with a girl/woman for the first time? __ __ years

21. How many different girls/women including your wife/wives have you had sex with during ….

21a. your lifetime? __ __ __

21b. last 12 months? __ __ __

21c. last 6 months? __ __ __

21d. last 30 days? __ __ __

22. Have you had sexual intercourse in the past 6 months? 1 = Yes 2 = No 29 = RE

23. How long since you last had sexual intercourse?

__ __ days

__ __ weeks

__ __ months

__ __ years

24. How many times have you had sexual intercourse including with your wife and any other partner in:

24a. Past 7 days __ __

24b. Past 30 days __ __

25. In the past 6 months, have you ever:

**25a. Had sex in a situation in which money or gifts were exchanged?**	1 = Yes 2 = No 29 = RE
**25b. Had vaginal sex with a woman while she was menstruating?**	1 = Yes 2 = No 29 = RE
**25c. Had sex with 2 or more partners during the same 30-day period?**	1 = Yes 2 = No 29 = RE
**26. Have you ever had sex with 2 or more partners during the same 30-day period?**	1 = Yes 2 = No 29 = RE

27. The last time you had sex..

**27a. Had you been drinking alcohol?**	1 = Yes 2 = No 29 = RE
**27b. Did you use a condom?**	1 = Yes 2 = No 29 = RE
**27c. Was it with…**	1 = your wife 2 = your regular partner<br>3 = non-regular partner 4 = sex worker<br>28 = DK 29 = RE
**28. How often have you placed your tongue on or licked the vagina of your sex partner?**	1 = Never 2 = Rarely 3 = Sometimes<br>4 = Often 5 = Always 28 = DK 29 = RE
**29. How often have you stuck your penis in the rectum (anus) of your partner?**	1 = Never 2 = Rarely 3 = Sometimes<br>4 = Often 5 = Always 28 = DK 29 = RE
**30. How often have you had sex with a woman the same day you met her?**	1 = Never 2 = Rarely 3 = Sometimes<br>4 = Often 5 = Always 28 = DK 29 = RE
**31. How often have you had sex after applying any substances (e.g., herbs, creams, lubricants, etc.) on your penis?**	1 = Never 2 = Rarely 3 = Sometimes<br>4 = Often 5 = Always 28 = DK 29 = RE
**31a. If ever, what substances?**	________________________________
**32. How often have you had sex when your partner has used substances on or in her vagina?**	1 = Never 2 = Rarely 3 = Sometimes<br>4 = Often 5 = Always 28 = DK 29 = RE
**32a. If ever, what substances?**	________________________________
**33. Do you prefer to have sexual intercourse when your partner's vagina is …..** (*read options*)	1 = Dry during intercourse<br>2 = Wet during intercourse<br>3 = No preference

If dry, in your experience, what does a woman do to make her vagina dry?

**33a. Wipes vagina with a towel/cloth**	1 = Yes 2 = No 28 = DK 29 = RE
**33b. Uses herbs and powders**	1 = Yes 2 = No 28 = DK 29 = RE
**33c. Uses commercially available products (e.g., antiseptic, soap, etc.)**	1 = Yes 2 = No 28 = DK 29 = RE
**34. Have you ever had a sexual relationship with a boy or man?**	1 = Yes 2 = No 29 = RE<br>No →(Go to 39)
**35. If yes, how many different boys/men?**	__ __
**36. Have you stuck your penis in the rectum (anus) of another man?**	1 = Yes 2 = No 29 = RE
**37. Have you had a penis stuck in your rectum (anus)?**	1 = Yes 2 = No 29 = RE
**38. Have you had a man ejaculate in your mouth?**	1 = Yes 2 = No 29 = RE

Section 4: Alcohol and Drug Use

**39. Did you have drinks containing alcohol like beer, spirits, chang'aa, busaa, etc. in the last four weeks?**	1 = Yes 2 = No 28 = DK 29 = RE<br>No →(Go to 42)
**40. How often did you have drinks containing alcohol in the last four weeks? Would you say… ?**	1 = At least once a day<br>2 = At least once a week<br>3 = Less than once a week
**41. During the last 6 months, how often have you had sex after or while you have been drinking?**	1 = Never 2 = Sometimes 3 = Often<br>4 = Always 28 = DK 29 = RE
**42. Some people have tried a range of different types of drugs. Which of the following, if any, have you tried?**	
**42a. Bhang / njaga**	1 = Yes 2 = No
**42b. Mandrax**	1 = Yes 2 = No
**42c. Valium**	1 = Yes 2 = No
**42d. Glue**	1 = Yes 2 = No
**42e. Miraa**	1 = Yes 2 = No
**42f. Kuber**	1 = Yes 2 = No
**42g. Other (specify)**	________________________________
**43. Some people have tried injecting drugs using a syringe. Have you ever injected drugs (not for medical reasons)?**	1 = Yes 2 = No 28 = DK 29 = RE

44. During the last 6 months, how often have you had sex while or after you have used some of these drugs? 1 = Never 2 = Sometimes 3 = Often 4 = Always 28 = DK 29 = RE

Section 5: Condom Use

45. Have you ever used a condom? 1 = Yes 2 = No 28 = DK 29 = RE
No →(Go to 49)

46. Have you ever experienced a problem with using condoms? 1 = Yes 2 = No 28 = DK 29 = RE

If yes, what kind of problem:

**46a. Did not know how to use condom**	1 = Yes 2 = No
**46b. Condom broke**	1 = Yes 2 = No
**46c. Condom slipped during the intercourse**	1 = Yes 2 = No
**46d. Condom was too large**	1 = Yes 2 = No
**46e. Condom was too small**	1 = Yes 2 = No
**46f. Other (specify)**	________________________

47. In the last 6 months, do you think it has been easy or difficult for you to obtain condoms? 1 = Very easy 2 = Somewhat easy 3 = Somewhat difficult 4 = Very difficult 28 = DK 29 = RE

48. Do you currently have a condom with you? 1 = Yes 2 = No 29 = RE

Section 6: Reproductive Health History

"Now I would like to ask you some questions about your reproductive health. Some men experience pain during urination, have discharge from the penis, or have sores around their genitals."

49. During the last 6 months, have you experienced:

**49a. Painful urination**	1 = Yes 2 = No
**49b. Frequent urination**	1 = Yes 2 = No
**49c. Sores around your genitals**	1 = Yes 2 = No
**49d. Discharge from your penis**	1 = Yes 2 = No
**49e. Difficulty passing urine (need to wait a long time until urine comes)**	1 = Yes 2 = No
**49f. Pain during intercourse**	1 = Yes 2 = No
**49g. Bleeding during intercourse**	1 = Yes 2 = No
**49h. Lower abdomen pain**	1 = Yes 2 = No
**49i. Genital warts**	1 = Yes 2 = No

50. If yes on any item in 49: Did you have sex while suffering from these symptoms? 1 = Yes 2 = No 28 = DK 29 = RE

51. Has a doctor or other health professional EVER told you that you had a sexually transmitted infection? Yes = 1 No = 2 28 = DK 29 = RE

52a. If yes, do you remember which STI? ________________________________

52. Did you have a sexually transmitted infection in past 6 months? 1 = Yes 2 = No 28 = DK 29 = RE

53. Have you EVER received treatment for a sexually transmitted infection? 1 = Yes 2 = No 28 = DK 29 = RE

53a. If yes, how many times? _____________

53b. Were you given an injection for treatment? 1 = Yes 2 = No

53c. Were you treated for a sexually transmitted infection in past 6 months? 1 = Yes 2 = No

Section 7: Sexual function and satisfaction

54. During the last 6 months, has there ever been a period of two weeks or more when you…….

54a. Lacked interest in sex? 1 = Yes 2 = No 28 = DK 29 = RE

54b. Were unable to come to a climax (experience an orgasm)? 1 = Yes 2 = No 28 = DK 29 = RE

54c. Came to a climax (had an orgasm) too quickly? 1 = Yes 2 = No 28 = DK 29 = RE

54d. Experienced pain during intercourse? 1 = Yes 2 = No 28 = DK 29 = RE

54e. Did not find sex pleasurable (even if it wasn't painful)? 1 = Yes 2 = No 28 = DK 29 = RE

54f. Had trouble achieving or maintaining erection? 1 = Yes 2 = No 28 = DK 29 = RE

55. In the past 6 months, how often during sex did your penis get sore? 1 = Never 2 = Rarely 2 = Sometimes 3 = Often 4 = Always 28 = DK 29 = RE

56. In the past 6 months, how often during sex did the skin of your penis get scratches, cuts, or abrasions? 1 = Never 2 = Rarely 2 = Sometimes 3 = Often 4 = Always 28 = DK 29 = RE

57. In the past 6 months, how often during or after sex did the skin of your penis bleed? 1 = Never 2 = Rarely 2 = Sometimes 3 = Often 4 = Always 28 = DK 29 = RE

58. Over the past 6 months, how would you generally rate your satisfaction with sexual intercourse? 1 = Very dissatisfied 2 = Dissatisfied

	3 = Satisfied	4 = Very satisfied
	28 = DK	29 = RE

More specifically, how satisfied are you with…

**58a. Level of sexual desire**	1 = Very dissatisfied	2 = Dissatisfied
	3 = Satisfied	4 = Very satisfied
	28 = DK	29 = RE
**58b. Getting erections**	1 = Very dissatisfied	2 = Dissatisfied
	3 = Satisfied	4 = Very satisfied
	28 = DK	29 = RE
**58c. Maintaining erections**	1 = Very dissatisfied	2 = Dissatisfied
	3 = Satisfied	4 = Very satisfied
	28 = DK	29 = RE
**58d. Ease of insertion of your penis into your partner's vagina**	1 = Very dissatisfied	2 = Dissatisfied
	3 = Satisfied	4 = Very satisfied
	28 = DK	29 = RE
**58e. Interval between erections**	1 = Very dissatisfied	2 = Dissatisfied
	3 = Satisfied	4 = Very satisfied
	28 = DK	29 = RE
**58f. Ease in ejaculation**	1 = Very dissatisfied	2 = Dissatisfied
	3 = Satisfied	4 = Very satisfied
	28 = DK	29 = RE
**58g. Level of pain on intercourse**	1 = Very dissatisfied	2 = Dissatisfied
	3 = Satisfied	4 = Very satisfied
	28 = DK	29 = RE
**58h. Spouse/partner opinion about my sexual performance**	1 = Very dissatisfied	2 = Dissatisfied
	3 = Satisfied	4 = Very satisfied
	28 = DK	29 = RE

Section 8: Perception of Risk and Sexual Activity

**59. In the past 6 months, do you believe your sexual activity has:**	1 = Decreased in frequency →Go to 60<br>2 = Increased in frequency →Go to 61<br>3 = Stayed the same →Go to 62<br>28 = DK →Go to 62<br>29 = RE →Go to 62
**60. What are some of the reasons your sexual activity has decreased in the last 6 months?**	
**60a. Fear of HIV/STIs**	1 = Yes 2 = No
**60b. Fewer opportunities to have sex**	1 = Yes 2 = No
**60c. Influences from the media, community, friends, etc.**	1 = Yes 2 = No
**60d. Personal decision to reduce sexual activity**	1 = Yes 2 = No
**60e. Other (specify)**	________________________
**61. What are some of the reasons your sexual activity has increased in the last 6 months?**	
**61a. Increase in sexual desire**	1 = Yes 2 = No
**61b. More opportunities to have sex (more partners are available and willing)**	1 = Yes 2 = No
**61c. Influences from the media, community, friends, etc.**	1 = Yes 2 = No
**61d. Circumcision**	1 = Yes 2 = No
**61e. Other (specify)**	________________________
**62. What do you think are your chances of getting HIV/AIDS?**	1 = No chance at all<br>2 = Small chance<br>3 = Moderate chance →Go to 64<br>4 = Great chance →Go to 64<br>5 = Don't know →Go to 65
**63. Why do you think that you have no chance at all or small chance of getting HIV/AIDS?**	When answered, go to 65
**63a. Am not having sex**	1 = Yes 2 = No
**63b. Always use condoms**	1 = Yes 2 = No
**63c. Have only one partner**	1 = Yes 2 = No

63d. Limit the number of partners 1 = Yes 2 = No

63e. Partner is faithful to me 1 = Yes 2 = No

63f. Circumcision 1 = Yes 2 = No

63g. Other (specify) ____

64. Why do you think that you have a moderate chance or a great chance of getting HIV/AIDS?

64a. Do not use condoms 1 = Yes 2 = No

64b. Have more than 1 sexual partner 1 = Yes 2 = No

64c. Partner has other partners 1 = Yes 2 = No

64d. Homosexual contacts 1 = Yes 2 = No

64e. Had blood transfusion/injection 1 = Yes 2 = No

64f. I was circumcised 1 = Yes 2 = No

64g. Other (specify) ____________________________________

65. Which activity, in your opinion, puts a person at a higher risk of HIV?

65a. Vaginal or anal intercourse?

1 = Unprotected vaginal intercourse is higher risk
2 = Unprotected anal intercourse is higher risk
3 = Both have same risk of HIV
28 = DK 29 = RE

65b. Dry (when your partner dries her vagina with cloth or substances) or wet sex??

1 = Unprotected wet sex is higher risk
2 = Unprotected dry sex is higher risk
3 = Both have same risk of HIV
28 = DK 29 = RE

66. Do you agree with following statements?

66a. It takes a lot of effort to keep your sexual behavior safe. 1 = Agree 2 = Not sure 3 = Disagree

66b. You feel tired of always having to monitor your sexual behavior. 1 = Agree 2 = Not sure 3 = Disagree

66c. When you are high or drunk, you are more likely to have sex with people other than your regular partner. 1 = Agree 2 = Not sure 3 = Disagree

66d. When you are high or drunk, you are more likely to have sex without a condom. 1 = Agree 2 = Not sure 3 = Disagree

Section 9: Beliefs about sex and sexuality

67. Please tell me whether you agree or disagree with the following statements.

**67a. Men can enjoy sex even with partners that they don't love**	1 = Strongly agree	2 = Agree	3 = Not sure
	4 = Disagree	5 = Strongly disagree	29 = RE
**67b. A "real man" is ready for sex at any time**	1 = Strongly agree	2 = Agree	3 = Not sure
	4 = Disagree	5 = Strongly disagree	29 = RE
**67c. Women have greater control over sexual desires than men**	1 = Strongly agree	2 = Agree	3 = Not sure
	4 = Disagree	5 = Strongly disagree	29 = RE
**67d. Sex is an important part of life**	1 = Strongly agree	2 = Agree	3 = Not sure
	4 = Disagree	5 = Strongly disagree	29 = RE
**67e. Inability to perform sexually is a source of sadness for partners**	1 = Strongly agree	2 = Agree	3 = Not sure
	4 = Disagree	5 = Strongly disagree	29 = RE
**67f. The quality of an erection is what satisfies women**	1 = Strongly agree	2 = Agree	3 = Not sure
	4 = Disagree	5 = Strongly disagree	29 = RE
**67g. A real man has sex very frequently**	1 = Strongly agree	2 = Agree	3 = Not sure
	4 = Disagree	5 = Strongly disagree	29 = RE
**67h. Women who are not sexually attractive can not be sexually satisfied**	1 = Strongly agree	2 = Agree	3 = Not sure
	4 = Disagree	5 = Strongly disagree	29 = RE
**67i. I am happy with my physical appearance**	1 = Strongly agree	2 = Agree	3 = Not sure
	4 = Disagree	5 = Strongly disagree	29 = RE

Section 10: Beliefs about circumcision

**68. Is it easier to keep your penis clean if you are…..?**	1 = Circumcised	2 = Uncircumcised	
	3 = No difference	28 = DK	29 = RE
**69. It is easier to get a disease from a woman if you are …?**	1 = Circumcised	2 = Uncircumcised	
	3 = No difference	28 = DK	29 = RE

**70. It is easier to get AIDS if you are …?**	1 = Circumcised 2 = Uncircumcised<br>3 = No difference 28 = DK 29 = RE
**71. Men enjoy sex more if they are …?**	1 = Circumcised 2 = Uncircumcised<br>3 = No difference 28 = DK 29 = RE
**72. Most women enjoy sex more with men who are …?**	1 = Circumcised 2 = Uncircumcised<br>3 = No difference 28 = DK 29 = RE
**73. Men who are ….. are more promiscuous**	1 = Circumcised 2 = Uncircumcised<br>3 = No difference 28 = DK 29 = RE
**74. Please state your opinion on circumcision:**	
**74a. Now that circumcision is available, HIV is a less serious threat than it used to be.**	1 = Agree 2 = Not sure 3 = Disagree
**74b. Now that circumcision is available, condom use during sex is less necessary.**	1 = Agree 2 = Not sure 3 = Disagree
**74c. Now that circumcision is available, I am less worried about HIV infection.**	1 = Agree 2 = Not sure 3 = Disagree
**74d. Now that circumcision is available, I am more likely to have more than one sexual partner.**	1 = Agree 2 = Not sure 3 = Disagree
**74e. Now that circumcision is available, I am more willing to take a chance of getting infected or infecting someone else with HIV.**	1 = Agree 2 = Not sure 3 = Disagree
**74f. Now that circumcision is available, someone who is HIV positive doesn't need to worry as much about condom use.**	1 = Agree 2 = Not sure 3 = Disagree
**74g. Now that circumcision is available, you are more likely to have sex without a condom.**	1 = Agree 2 = Not sure 3 = Disagree

Section 11: Hygiene

**75. How often do you normally wash your penis?**	1 = Once a month or less 2 = Several times a month<br>3 = Weekly 4 = Several times a week<br>5 = Daily 6 = Don't wash penis
**76. The last time you had vaginal intercourse, did you wash your genitals immediately after sex?**	1 = Yes 2 = No 28 = DK 29 = RE
**What did you use to clean your penis?**	
**76a. Cloth/rag without water**	1 = Yes 2 = No 28 = DK 29 = RE
**76b. Water**	1 = Yes 2 = No 28 = DK 29 = RE

76c. Soap and water 1 = Yes 2 = No 28 = DK 29 = RE

76d. Herbs 1 = Yes 2 = No 28 = DK 29 = RE

76e. Other (specify) ________________________________

76f. How long it until you washed your penis after last vaginal intercourse? _______hrs _____ min

77. Have you ever put or applied substance(s) on head of your penis for any reason?
1 = Yes
2 = No 28 = DK 29 = RE →Go 78

77a. If yes, why did you apply those substances?
1 = For cleaning / killing germs
2 = Removing bad odor
3 = Contraception
4 = To cure itching
5 = After sex to clean the penis
6 = Before sex for initiation/preparation
7 = To treat ulcers
8 = To protect myself against disease/infection
9 = To protect partner from disease/infection
29 = RE

Section 12: HIV testing and Counseling

78. Have you ever been tested for HIV? 1 = Yes 2 = No 28 = DK 29 = RE

79. When you were tested for HIV, did you receive information on how to reduce your risk of acquiring or transmitting HIV? 1 = Yes 2 = No 28 = DK 29 = RE

80. How many times have you been tested for HIV? __ __

81. When was the last time you were tested?
1 = less than one year ago 2 = 1-2 years ago
3 = more than 2 years ago

82. I am going to ask you about the result of your HIV test, but you may refuse to answer if you wish. What was the result of your last HIV test?
1 = Positive 2 = Negative (go to 85)
3 = Did not get the result (go to 85)
29 = RE (go to 85)

83. Do you go to a clinic to get HIV care? 1 = Yes 2 = No 28 = DK 29 = RE

84. Are you on antiretroviral therapy (ART)? 1 = Yes 2 = No 28 = DK 29 = RE

Section 13: Circumcision

**85. Are you circumcised?**	1 = Yes		
	2 = No	→ Go to 102	
**86. Where were you circumcised?**	1 = In this clinic		
	2 = Other (specify)______________________		
**87. How long ago were you circumcised?**	__ __ days		
	__ __ weeks		
	__ __ months		
**88. How satisfied are you ….**			
**88a. With the way your circumcision was carried out?**	1 = Very satisfied	2 = Somewhat satisfied	
	3 = No opinion	4 = Somewhat dissatisfied	
	5 = Very dissatisfied	28 = DK	29 = RE
**88b. With the appearance of your penis?**	1 = Very satisfied	2 = Somewhat satisfied	
	3 = No opinion	4 = Somewhat dissatisfied	
	5 = Very dissatisfied	28 = DK	29 = RE
**88c. With your sexual performance?**	1 = Very satisfied	2 = Somewhat satisfied	
	3 = No opinion	4 = Somewhat dissatisfied	
	5 = Very dissatisfied	28 = DK	29 = RE
**89. Have you had an erection since the circumcision?**	1 = Yes	2 = No	29 = RE
**89a. If yes, do your erections feel normal?**	1 = Yes	2 = No	29 = RE
**89a.1 If no, why not?**			
**It hurts**	1 = Yes	2 = No	29 = RE
**it bleeds**	1 = Yes	2 = No	29 = RE
**it feels tight/stretched**	1 = Yes	2 = No	29 = RE
**89b. If yes to 90, compared to before you were circumcised, how hard are your erections**	1 = Hard	2 = Less hard	3 = About the same
	29 = RE		
**90. Other than while you were healing, have you ever avoided sex because of being circumcised?**	1 = Yes 2 = No 28 = DK 29 = RE		

**91. Have you had intercourse since the operation?**	1 = Yes<br>2 = No 29 = RE →Go to 97f
**92. How soon after surgery did you first have sexual intercourse?**	__ __ hours<br>__ __ days<br>__ __ weeks<br>__ __ months
**93. Have you used condoms after becoming circumcised?**	1 = Yes<br>2 = No 29 = RE →Go to 95
**94. Did you find that it was easier to use condoms after circumcision, compared to when you were uncircumcised?**	1 = Easier after circumcision<br>2 = Easier before circumcision<br>3 = About the same<br>4 = Did not use condoms before circumcision<br>28 = DK<br>29 = RE
**95. Did you find that sex was more enjoyable before circumcision or after circumcision?**	1 = More enjoyable before circumcision<br>2 = More enjoyable after circumcision<br>3 = No difference<br>28 = DK<br>29 = RE
**96. Do you think that sex was more enjoyable before your circumcision or after circumcision for your partner(s)?**	1 = More enjoyable for partners before MC<br>2 = More enjoyable for partners after MC<br>3 = No difference<br>28 = DK<br>29 = RE

97. Compared to before you were circumcised:

**97a. How sensitive would you say your penis is?**	1=Much more<br>3=About the same<br>5=Much less	2=Somewhat more<br>4=Somewhat less<br>28=DK 29=RE
**97b. How easily do you think you reach orgasm during sex?**	1=Much more<br>3=About the same<br>5=Much less	2=Somewhat more<br>4=Somewhat less<br>28=DK 29=RE

97c. How often do you have sex?

1=Much more 2=Somewhat more
3=About the same 4=Somewhat less
5=Much less 28=DK 29=RE

97d. Do you experience more, less, or about the same amount of scratches, tears, or cuts?

1=Much more 2=Somewhat more
3=About the same 4=Somewhat less
5=Much less 6= Did not experience scratches before
28=DK 29=RE

97e. Do you ejaculate earlier than you wanted?

1=Much more 2=Somewhat more
3=About the same 4=Somewhat less
5=Much less 6= Did not experience early ejaculation before
28=DK 29=RE

97f. How protected do you feel against sexual diseases?

1=Much more 2=Somewhat more
3=About the same 4=Somewhat less
5=Much less 28=DK 29=RE

97g. Is your aim during urination better, worse, or about the same?

1=Much better 2=Somewhat better
3=About the same 4=Somewhat worse
5=Much worse 28=DK 29=RE

98. Has circumcision changed your sexual behavior in any way?

1 = Yes 2 = No 28 = DK 29 = RE

99. Since you've been circumcised, how attracted are women to you?

1=Much more 2=Somewhat more
3=About the same 4=Somewhat less
5=Much less 28=DK 29=RE

100. Other than your sex partners, is anyone in our family aware that you have been circumcised?

1 = Yes 2 = No 28 = DK 29 = RE

100a. If yes, overall, what is their reaction?

1=Very pleased
2=Somewhat pleased
3=Neutral or expressed no opinion
4=Somewhat displeased
5=Very displeased
28=DK 29=RE

101. Have any of your sex partners been aware that

1 = Yes 2 = No 28 = DK 29 = RE

you have been circumcised?

**101a. If yes, were they pleased or displeased?**	1=Very pleased<br>2=Somewhat pleased<br>3=Neutral or expressed no opinion<br>4=Somewhat displeased<br>5=Very displeased<br>28=DK 29=RE

102. What are some of the considerations that would/did encourage you to become circumcised?

**102a. Improved hygiene**	1 = Yes	2 = No	28 = DK	29 = RE
**102b. Protection from STI/HIV**	1 = Yes	2 = No	28 = DK	29 = RE
**102c. Acceptability by other ethnic groups**	1 = Yes	2 = No	28 = DK	29 = RE
**102d. Sexual pleasure for self**	1 = Yes	2 = No	28 = DK	29 = RE
**102e. Sexual pleasure for partners**	1 = Yes	2 = No	28 = DK	29 = RE
**102f. No cost**	1 = Yes	2 = No	28 = DK	29 = RE
**102g. It was available in a local health facility**	1 = Yes	2 = No	28 = DK	29 = RE
**102h. Influence by friends / peers**	1 = Yes	2 = No	28 = DK	29 = RE
**102i. Other, specify**	________________			

103. What are some of the considerations that would/did discourage you from becoming circumcised?

**103a. Pain during/after the surgery**	1 = Yes	2 = No	28 = DK	29 = RE
**103b. Potential adverse events and complications**	1 = Yes	2 = No	28 = DK	29 = RE
**103c. Culture / Tradition / Religion**	1 = Yes	2 = No	28 = DK	29 = RE
**103d. Cost of procedure, including time off work**	1 = Yes	2 = No	28 = DK	29 = RE
**103e. Sexual pleasure for self**	1 = Yes	2 = No	28 = DK	29 = RE
**103f. Sexual pleasure for partners**	1 = Yes	2 = No	28 = DK	29 = RE
**103g. Nearest facility is far**	1 = Yes	2 = No	28 = DK	29 = RE
**103h. Long healing period**	1 = Yes	2 = No	28 = DK	29 = RE
**103i. Other, specify**	________________			

**104. Would you circumcise your son(s)?**	1 = Yes	2 = No	28 = DK	29 = RE
**104a. If yes, at what age?**	________________			

"Thank you very much for your cooperation. The information you provided is very helpful and we appreciate your time and assistance. Do you have any final questions or comments that you would like to share with me?"

"I will now ask you detailed information about every sexual relationship you had in past 6 months." Go to Form 03 Sexual History
